# NPAS3-regulated astrocyte mitochondrial bioenergetics is required for cognition

**DOI:** 10.1126/sciadv.adt2527

**Published:** 2026-06-17

**Authors:** Kateryna Murlanova, Ksenia Novototskaya-Vlasova, Shovgi Huseynov, Olga Pletnikova, Rebecca D. Howell, Yan Jouroukhin, Dong Won Kim, Adrian Eddy-Sulaiman Jenson, Juhyun Lee, Cheng Qin, Stephen R. Thompson, Vikyath Saraf, Michael J. Morales, Eduardo Cortes Gomez, Russell L. Margolis, Frederick C. Nucifora, Spencer R. Rosario, Andrew A. Pieper, Henry G. Withers, Samir Haj-Dahmane, Juhyun Kim, Mikhail V. Pletnikov

**Affiliations:** ^1^ Department of Physiology and Biophysics, Jacobs School of Medicine and Biomedical Sciences, State University of New York at Buffalo, Buffalo, NY, USA.; ^2^ Department of Pathology and Anatomical Sciences, Jacobs School of Medicine and Biomedical Sciences, State University of New York at Buffalo, Buffalo, NY, USA.; ^3^ Department of Pharmacology and Toxicology, Jacobs School of Medicine and Biomedical Sciences, State University of New York at Buffalo, Buffalo, NY, USA.; ^4^ Danish Research Institute of Translational Neuroscience (DANDRITE), Nordic EMBL Partnership for Molecular Medicine, Aarhus University, Aarhus, Denmark.; ^5^ Department of Biomedicine, Aarhus University, Aarhus, Denmark.; ^6^ Department of Psychiatry and Behavioral Sciences, Johns Hopkins University School of Medicine, Baltimore, MD, USA.; ^7^ Emotion, Cognition & Behavior Research Group, Korea Brain Research Institute, Daegu, Republic of Korea.; ^8^ School of Medicine, Kyungpook National University, Daegu, Republic of Korea.; ^9^ Department of Biostatistics and Bioinformatics, Roswell Park Comprehensive Cancer Center, Buffalo, NY, USA.; ^10^ Department of Neurology, Johns Hopkins School of Medicine Baltimore, MD, USA.; ^11^ Department of Psychiatry, Department of Neurosciences, Department of Pathology, Case Western Reserve University and Institute for Transformative Molecular Medicine, Cleveland, OH, USA.; ^12^ Brain Health Medicines Center, Harrington Discovery Institute, University Hospitals, Cleveland Medical Center, Cleveland, OH, USA.; ^13^ Geriatric Psychiatry, GRECC, Louis Stokes VA Medical Center, Cleveland, OH, USA.

## Abstract

The basic helix-loop-helix transcription factor neuronal PAS (Per, Arnt, Sim) domain protein 3 (NPAS3) provides transcriptional regulation of metabolic pathways and is highly expressed in astrocytes. NPAS3 variants have been associated with cognitive dysfunction under several neuropsychiatric conditions, but the underlying brain cell type–specific mechanisms remain obscure. Here, we report that NPAS3 is a key regulator of mitochondrial bioenergetics in astrocytes in the mouse brain. Selective deletion of *Npas3* in mature astrocytes decreases expression of mitochondrial glutamate carrier 2 involved in glutamate oxidation, leading to reduced oxidative phosphorylation and lactate production in astrocytes. This deficit reduces intrinsic excitability, dendritic spine density, and excitatory synaptic transmission of medial prefrontal cortex (mPFC) pyramidal neurons. Mice with *Npas3*-deficient mPFC astrocytes exhibit impaired trace fear conditioning, which is rescued by lactate treatment. Thus, the present study demonstrates a mechanistic link between NPAS3-dependent astrocyte mitochondrial bioenergetics and cognitive function and provides insights for glia-targeting treatment of cognitive dysfunction in neuropsychiatric disease.

## Teaser

NPAS3 regulates astrocyte energy metabolism required for cognition, highlighting glial targets for neuropsychiatric disorders.

## INTRODUCTION

Astrocytes are increasingly recognized as playing a crucial role in brain metabolism and overall brain function (*1*–*3*). These glial cells are essential not only for maintaining the structural and functional integrity of neurons but also for supporting their metabolic needs. The metabolic processes occurring within astrocytes, particularly those involving mitochondrial function, are fundamental for providing neurons with the energy required to sustain critical functions such as action potential generation and synaptic transmission. Consequently, recent studies have demonstrated that modulating astrocyte activity can lead to changes in brain-wide physiology and behaviors (*4*–*8*).

Prior studies have shown that the basic helix-loop-helix (bHLH) transcription factor neuronal PAS (Per, Arnt, Sim) domain protein 3 (NPAS3), despite its name, is highly expressed in astrocytes (*9*). NPAS3-dependent transcriptional regulation of metabolic pathways has been suggested, including altered levels of nicotinamide adenine dinucleotide (oxidized form) [NAD^+^], glycolysis metabolites, pentose phosphate pathway components, and Krebs cycle intermediates (*10*). However, the underlying mechanisms remain unknown.

NPAS3 has been implicated in brain development, cognitive impairment, and psychiatric disorders in both humans and animal models. Genetic studies of a family affected with schizophrenia and intellectual disability suggested that haploinsufficiency of NPAS3 contributes to the pathogenesis of mental illness (*11*, *12*). In addition, NPAS3 was identified as a clinically important gene in copy number variation analyses of both autism spectrum disorder and schizophrenia (*13*). In our previous studies, we demonstrated that the NPAS3 Val304→Ile (V304I) single-nucleotide polymorphism leads to protein aggregation in mammalian cell systems, potentially contributing to mental illness (*14*, *15*). Animal models with altered *Npas3* expression have revealed cognitive impairment and psychiatric symptoms (*9*, *16*, *17*), alongside a reduction in dendritic spine density in neurons (*9*, *18*). However, the mechanisms of how astrocyte-enriched *Npas3* alterations affect astrocytic physiology, as well as consequently neuronal function and cognitive behavior, remain unclear.

This study uses a combination of RNA sequencing (RNA-seq) gene expression profiling, astrocyte-specific knockout (KO) of *Npas3*, in vitro and in vivo astrocyte metabolic assay, electrophysiological measurements, dendritic spine analysis, and cognitive tests to explore the functional relationship between *Npas3* dysfunction, astrocytic metabolism, and neuronal and behavioral alterations. Here, we report that *Npas3* is involved in astrocyte mitochondrial bioenergetics via regulation of the expression of mitochondrial glutamate carrier 2 (GC2), which is involved in glutamate oxidation, reduced expression of which leads to reduced oxidative phosphorylation (OXPHOS), lactate production, decreased neuronal excitability, and cognitive impairment that can be rescued with lactate treatment.

## RESULTS

### *Npas3* regulates the expression of GC2 in astrocytes

**Fig. 1.**
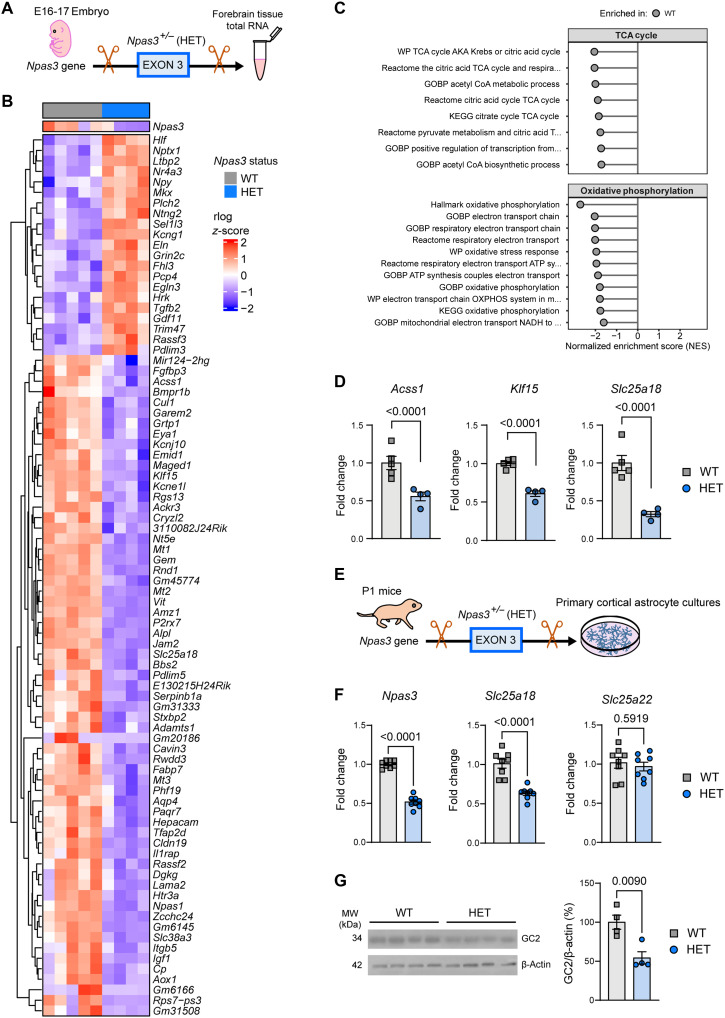
*Npas3* regulates metabolic processes via expression of GC2 in astrocytes. (**A**) Strategy used to obtain *Npas3^+/−^* (HET) embryonic forebrain tissue. (**B**) Row-wise scaled heatmap showing relative, normalized expression of differentially expressed genes (FDR-corrected *P* < 0.05) in forebrain tissue [embryonic day 16 (E16) to E17] from *Npas3^+/−^* (HET) (*n* = 4) compared to WT (*n* = 5) female mice. (**C**) GSEA showing energy metabolism processes enriched in the forebrain (E16 to E17) of WT compared with *Npas3^+/−^* (HET) female mice. All reported enrichment scores are significant (adjusted *P* < 0.05). (**D**) Normalized relative mRNA expression of *Acss1*, *Klf15*, and *Slc25a18* in forebrain tissue (E16 to E17) of WT and *Npas3^+/−^* (HET) female mice (*n* = 4 to 5 female mice; unpaired Student’s *t* test, two-tailed). (**E**) Strategy used to obtain *Npas3^+/−^* (HET) cortical astrocytes in primary culture. (**F**) Normalized levels of *Npas3*, *Slc25a18* (GC2), and *Slc25a22* (GC1) in WT and *Npas3^+/−^* (HET) primary cortical astrocyte cultures (*n* = 8 biologically independent cell culture preparations; unpaired Student’s *t* test, two-tailed). (**G**) Western blot against GC2 protein and normalized levels of GC2 in WT and *Npas3^+/−^* (HET) cortical astrocytes in primary culture. β-Actin was used as a loading control (*n* = 4 biologically independent cell culture preparations; unpaired Student’s *t* test, two-tailed). Data are presented as means ± SEM. MW, molecular weight; WP, WikiPathways; GOBP, gene ontology biological process.

To unravel the contributions of NPAS3 to bioenergetic processes, we performed RNA-seq expression profiling on bulk embryonic forebrain tissue of mice with intact *Npas3* [wild-type (WT); *n* = 5] or heterozygous deletion of *Npas3* [*Npas3^+/−^* (HET); *n* = 4] (Fig. 1A), as generated previously (*18*). Differential gene expression analysis of samples from *Npas3^+/−^* (HET) mice relative to WT samples revealed 21 significantly up-regulated genes and 63 significantly down-regulated genes [false discovery rate (FDR) < 0.05; Fig. 1B and data S1]. In support of previous RNA-seq studies (*9*, *19*), we observed a number of consistent changes in the expression of genes related to cellular energy metabolism in samples from *Npas3^+/−^-*deficient mice. The differentially expressed genes included those involved in metabolic reprogramming (e.g., *Idf1*, *Cavin3*, *Acss1*, and *Slc25a18*), amino acid metabolism, adenosine 5′-monophosphate–activated protein kinase, lipid and acetyl–coenzyme A (CoA) metabolism (e.g., *Fabp7* and *Klf15*), and insulin signaling (e.g., *Idf1*). Gene set enrichment analysis (GSEA) for mouse hallmark (MH) gene sets from the Mouse Molecular Signatures Database (MSigDB) identified OXPHOS as significantly enriched in the control samples (FDR < 0.05; data S2). We expanded this analysis to include curated canonical pathways [M2 : canonical pathways (CP) collection] and ontology (M5: gene ontology biological process collection) gene sets from MSigDB that further supported the consistent and significant enrichment of both OXPHOS and the citric acid cycle in control samples compared to *Npas3^+/−^* (Fig. 1C). To identify potential NPAS3 regulated genes contributing to these mitochondrial metabolic pathways, we filtered differentially expressed genes to include candidates that (i) contain E-box (CANNTG), a consensus site for NPAS3 (*20*) sequence(s) in their promoter regions; (ii) are predominantly expressed by astrocytes as evidenced across several publicly available transcriptomic databases (*21*–*25*), and (iii) are involved in mitochondrial function and/or energy metabolism as determined by comprehensive literature review and gene ontology classifications. In addition to the down-regulation of well characterized acetyl-CoA synthetase enzyme (*Acss1*) and a Krüppel-like transcription factor (*Klf15*) responsible for metabolic reprogramming (*26*, *27*), we identified the solute carrier family 25 member 18 (*Slc25a18*) as significantly down-regulated in samples from *Npas3^+/−^* (HET) mice (Fig. 1D). The *Slc25a18* gene encodes GC2, which, along with GC1 (*Slc25a22*), transports glutamate through the mitochondrial inner membrane to the matrix, allowing glutamate dehydrogenase to generate α-ketoglutarate to be used in the tricarboxylic acid (TCA) cycle (*28*, *29*). Astrocytes highly express both GC1 and GC2; GC2 is enriched in astrocyte endfeet where mitochondria are abundant (*30*, *31*). Thus, we chose GC2 as an NPAS3-regulated candidate to evaluate its role in astrocyte mitochondrial bioenergetics in the context of higher brain functions and neuropsychiatric disorders.

### Energy metabolism in *Npas3*-deficient astrocytes depends on GC2

To provide a more comprehensive evaluation of the role of GC2 in the energy metabolism in astrocytes, we used primary cortical astrocytes prepared from *Npas3^+/−^* (HET) mice (Fig. 1E). We first confirmed reduced *Npas3* and *Slc25a18* mRNA expression in primary cortical *Npas3^+/−^* astrocytes (Fig. 1F). Notably, loss of *Npas3* did not affect the expression of *Slc25a22* (GC1), another glutamate transporter in astrocytes (Fig. 1F), suggesting a selective reduction of GC2 levels in *Npas3*-deficient astrocytes. We also confirmed that *Slc25a18* protein product GC2 was decreased in primary cortical astrocytes (Fig. 1G).

**Fig. 2.**
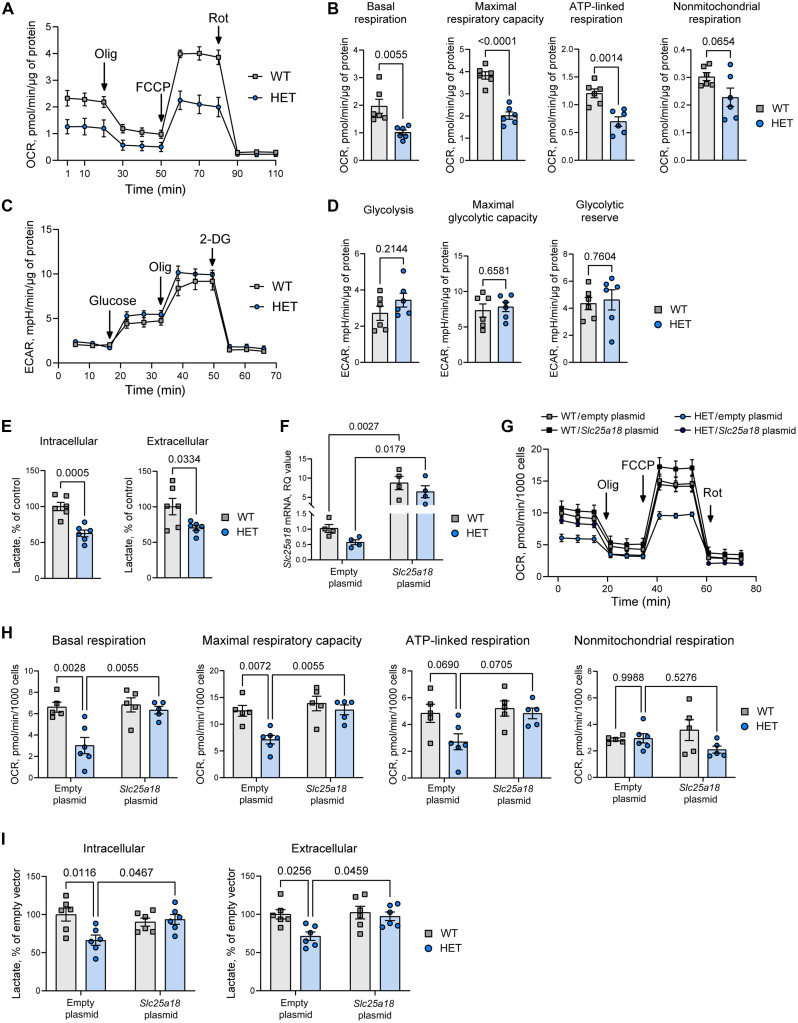
Deficiency of *Npas3* in astrocytes inhibits OXPHOS and lactate production rescued by GC2 overexpression. (**A**) OCR for WT and *Npas3^+/−^* (HET) cortical astrocytes in primary cell culture. Rot, rotenone; Olig, oligomycin. (**B**) Basal, maximum, ATP-linked respiration, and nonmitochondrial respiration in WT and *Npas3^+/−^* (HET) astrocytes (*n* = 6 biologically independent cell culture preparations; unpaired Student’s *t* test, two-tailed). (**C** and **D**) ECAR and calculated parameters (glycolysis, maximal glycolytic capacity, and glycolytic reserve) in WT and *Npas3^+/−^* (HET) cortical astrocytes in primary cell culture (*n* = 6 biologically independent cell culture preparations; unpaired Student’s *t* test, two-tailed). (**E**) Normalized levels of lactate in WT and *Npas3^+/−^* (HET) primary cortical astrocytes and culture medium (*n* = 6 biologically independent cell culture preparations; unpaired Student’s *t* test, two-tailed). (**F**) *Slc25a18* mRNA expression in primary cortical astrocytes following *Slc25a18* overexpression. *Slc25a18* mRNA was detected by quantitative real-time polymerase chain reaction (PCR) in WT and *Npas3^+/−^* (HET) primary cortical astrocytes transfected with either an empty plasmid (pCMV6-Entry) or the same plasmid encoding MYC-tagged mouse *Slc25a18* (GC2 protein) (*n* = 4 biologically independent cell culture preparations; two-way ANOVA, followed by Tukey post hoc test). RQ, relative quantification. (**G**) OCR for WT and *Npas3^+/−^* (HET) cortical astrocytes in primary cell culture, transfected with either an empty plasmid or a *Slc25a18* plasmid. (**H**) Basal, maximum, ATP-linked respiration, and nonmitochondrial respiration in WT and *Npas3^+/−^* (HET) astrocytes following *Slc25a18* overexpression (*n* = 5 to 6 biologically independent cell culture preparations; two-way ANOVA, followed by Tukey post hoc test). (**I**) Normalized levels of lactate in WT and *Npas3^+/−^* (HET) cortical astrocytes (intracellular) and cell culture medium (extracellular) following *Slc25a18* overexpression. Lactate levels were normalized to the values obtained from WT samples after transfection with the empty plasmid (vector) (*n* = 6 biologically independent cell culture preparations; two-way ANOVA, followed by Tukey post hoc test). Data are presented as means ± SEM.

Next, we analyzed the effects of *Npas3* deletion on oxygen consumption rate (OCR) and extracellular acidification rate (ECAR) using the Seahorse XF Cell Mito Stress and Glycolysis Stress tests. Compared to WT, *Npas3^+/−^* (HET) astrocytes exhibited significantly lower OCRs (Fig. 2A), indicating deficient mitochondrial OXPHOS. A roughly twofold decrease was observed in basal respiration, maximal respiratory capacity [measured after carbonyl cyanide *p*-trifluoromethoxyphenylhydrazone (FCCP) addition], and adenosine 5′-triphosphate (ATP)–linked respiration (basal respiration minus respiration after oligomycin addition) of *Npas3^+/−^* (HET) astrocytes (Fig. 2B). No genotype-dependent changes were observed in nonmitochondrial respiration after injection of rotenone (Fig. 2B). The *Npas3*-mediated effects on mitochondrial function were not linked to significant changes in expression levels of the respiratory complexes CII, CIII, CIV, and CV (fig. S1, A and B).

In contrast to OCR, no significant alterations in ECAR were observed (Fig. 2C), suggesting comparable glycolytic function between WT and *Npas3^+/−^* (HET) astrocytes. *Npas3^+/−^* (HET) and WT astrocytes demonstrated comparable basal ECAR levels. No difference was observed after glucose was added, suggesting that astrocytes can increase glycolysis when needed, regardless of the expression level of *Npas3*. When oligomycin was introduced, which inhibits aerobic respiration and forces cells to depend on glycolysis for energy, both *Npas3^+/−^* (HET) and WT astrocytes showed similar rates of acidification, further indicating their capacity for enhanced glycolysis. In addition, the ECAR after treatment with 2-deoxyglucose (2-DG), which reflects nonglycolytic acidification, was similar between the two groups. No genotype-dependent changes were observed in the glycolysis (acidification after glucose addition minus basal acidification), maximal glycolytic capacity (measured after oligomycin addition), and glycolytic reserve (acidification after oligomycin minus acidification after glucose addition) (Fig. 2D). In agreement with ECAR data, no changes in proton efflux rate (PER) were observed (fig. S2A). *Npas3^+/−^* (HET) and WT astrocytes showed similar levels of baseline PER and compensatory PER after oligomycin addition (fig. S2, B and C). Since PER assesses glycolysis-associated proton production and export while minimizing mitochondrial contributions, these data suggest that glycolysis-associated proton efflux is not altered in *Npas3*-deficient astrocytes. Further, stable isotope tracing with [U-^13^C] glucose did not show any statistically significant differences in consumption/utilization of glycolytic metabolites between WT and HET groups, confirming that there are no differences associated with glycolysis (fig. S3).

We next evaluated the levels of the major glycolytic (*32*) and glutamate oxidation (*33*) product, lactate, in *Npas3*-deficient astrocytes. We detected significantly lower intracellular and extracellular levels of lactate in *Npas3*-deficient astrocytes. An approximately 1.5-fold decrease in lactate levels was observed in cell pellets and culture medium of *Npas3^+/−^* (HET) astrocytes compared to WT astrocytes (Fig. 2E).

To further establish a functional link between GC2, OXPHOS, and lactate production in astrocytes, we overexpressed GC2 in the *Npas3*-deficient astrocytes. *Npas3^+/−^* (HET) and WT astrocytes were transfected with an empty plasmid or a *Slc25a18* (GC2) overexpression plasmid (Fig. 2F), and OCR was measured. We found that *Npas3^+/−^* (HET) astrocytes showed an increase in OCR after transfection with the *Slc25a18* overexpression plasmid compared to the empty vector (Fig. 2G). The decrease in mitochondrial respiration (basal respiration, maximal respiratory capacity, and ATP-linked respiration) was restored to levels observed in WT astrocytes (Fig. 2H). In addition, GC2 overexpression in HET astrocytes led to restoration of intracellular and extracellular levels of lactate in WT astrocytes (Fig. 2I). Together, our data indicate that reduced expression of GC2 in *Npas3*-deficient astrocytes leads to decreased OXPHOS and lactate production and release by astrocytes.

### Astrocyte-specific *Npas3* deficiency in vivo reduces energy metabolism

**Fig. 3.**
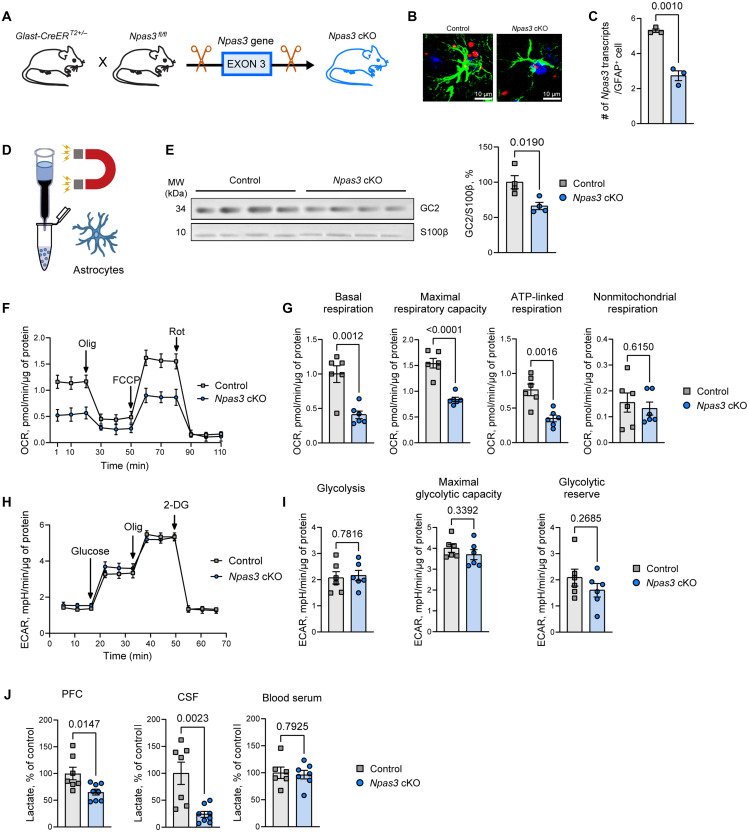
In vivo astrocyte-restricted deletion of *Npas3* leads to altered energy metabolism. (**A**) Strategy used to generate astrocyte-specific *Npas3* cKO mice. (**B**) Representative confocal images of dual BaseScope ISH for *Npas3* mRNA (red punctate dots) and glial fibrillary acidic protein (GFAP) immunofluorescence (IF) (green). Sections were counterstained with 4′,6-diamidino-2-phenylindole (DAPI) fluorescent dye (blue) to disclose tissue morphology, i.e., the cell nuclei. All images were obtained using a 63× objective. (**C**) Quantitative analysis of *Npas3* mRNA expression in the PFC. Data points represent individual animals. An average of 150 of astrocytes per the mouse PFC were analyzed (*n* = 3 male mice per group; unpaired Student’s *t* test, two-tailed). (**D**) Strategy to immunomagnetically isolate astrocytes from the PFC. (**E**) Western blot images of GC2 protein expression and normalized levels of GC2 in control and *Npas3* cKO astrocytes. S100β was used as a loading control (*n* = 4 male mice per group; unpaired Student’s *t* test, two-tailed). (**F** and **G**) OCR and the calculated parameters (basal respiration, maximal respiratory capacity, ATP-linked respiration, and nonmitochondrial respiration) of astrocytes of control and *Npas3* cKO mice (*n* = 6 male mice per group; unpaired Student’s *t* test, two-tailed). (**H** and **I**) ECAR and the calculated parameters (glycolysis, maximal glycolytic capacity, and glycolytic reserve) of astrocytes of control and *Npas3* cKO mice (*n* = 6 male mice per group; unpaired Student’s *t* test, two-tailed). (**J**) Normalized levels of lactate in the PFC, cerebrospinal fluid (CSF), and blood serum in control and *Npas3* cKO mice (*n* = 7 to 8 male mice per group; unpaired Student’s *t* test, two-tailed). Data are presented as means ± SEM.

To investigate the role of NPAS3 in astrocyte bioenergetics in vivo, we crossed *Npas3^fl/fl^* mice (*18*) with glutamate-aspartate transporter (GLAST)::CreER^T2^ mice (*34*) to generate GLAST::CreER^T2^ × *Npas3fl^/+^* [*Npas3* conditional KO (cKO) mice]. Cre-negative male and female littermates carrying the floxed *Npas3* allele (*Npas3^fl/+^*) obtained from the same GLAST::CreER^T2^ × *Npas3^fl/fl^* breeding were used as controls (Fig. 3A). All mice were treated with tamoxifen and were on the C57BL/6J background. We first validated astrocyte-specific reduction of *Npas3* expression. BaseScope in situ hybridization (ISH) combined with immunostaining was used to assess the expression of *Npas3* mRNA in the prefrontal cortex (PFC), which exhibits high specificity and efficiency of GLAST-CreER^T2^–mediated recombination (*35*) and NPAS3 expression (*9*, *19*). We confirmed a 50% decrease in *Npas3* mRNA expression in astrocytes of the PFC in *Npas3* cKO mice compared to control mice (Fig. 3, B and C).

Astrocytes were immunomagnetically isolated from the whole brain using anti-astrocyte cell surface antigen-2 (anti–ACSA2) microbeads (Fig. 3D), optimized to yield pure astrocytes as validated by the expression of astrocyte-specific, but not neuronal- or microglial-specific factors (fig. S4). Consistent with our in vitro results, we detected lower levels of GC2 protein in resting membrane potential astrocytes immunomagnetically isolated from the cortex (Fig. 3E), suggesting reduced glutamate oxidative metabolism in astrocyte mitochondria. Similar to the OCR findings from primary astrocytes, *Npas3*-deficient cortical astrocytes exhibited decreased mitochondrial OXPHOS (Fig. 3F) as characterized by a twofold reduction in the basal respiration, maximal respiratory capacity, and ATP-linked respiration without any changes in nonmitochondrial respiration (Fig. 3G). In a similar vein, *Npas3* cKO and control astrocytes exhibited comparable levels of ECAR (Fig. 3H). No genotype-dependent changes were observed in the glycolysis, maximal glycolytic capacity, and glycolytic reserve (Fig. 3I).

Given the previously established functional link between GC2 and lactate production in primary astrocytes (Fig. 2E), we next measured lactate levels in the PFC, cerebrospinal fluid (CSF), and blood serum of *Npas3* cKO mice. Compared with control mice, *Npas3* cKO mice showed decreased lactate levels in the PFC and CSF but not in blood serum (Fig. 3J). The results indicate that in vivo astrocyte-restricted deletion of *Npas3* leads to reduced expression of astrocytic GC2, decreased astrocytic OXPHOS, and diminished levels of lactate in the central nervous system.

### Astrocytic *Npas3* deficiency reduces intrinsic excitability in medial PFC pyramidal neurons

**Fig. 4.**
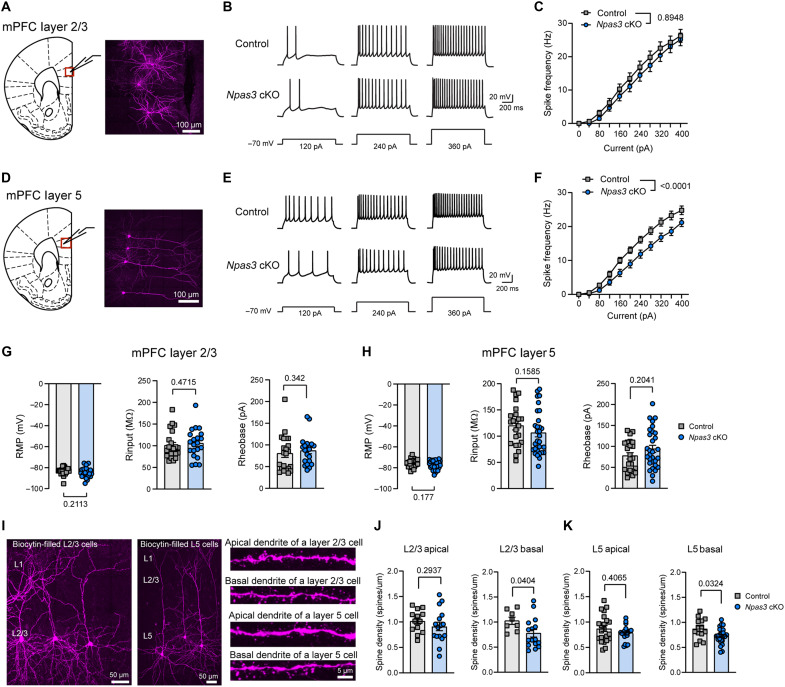
*Npas3* cKO mice exhibit decreased intrinsic excitability and dendritic spine density in the mPFC. (**A**) Experimental configuration (left) and example (right) of biocytin-filled L2/3 pyramidal neurons. (**B**) Representative voltage. (**C**) Spike frequency summary for L2/3 neurons (control, 21 cells from four male mice; cKO, 21 cells from six male mice; repeated-measures two-way ANOVA). (**D**) Experimental configuration and example of biocytin-filled L5 pyramidal neurons. (**E**) Representative voltage traces. (**F**) Spike frequency summary for L5 neurons (control, 23 cells from five male mice; cKO, 29 cells from nine male mice; repeated-measures two-way ANOVA). (**G**) Resting membrane potential (RMP). Summary graphs for basal membrane properties of L2/3 neurons (control, 22 cells from four male mice; cKO, 21 cells from six male mice). (**H**) Summary graphs for basal membrane properties of L5 neurons (control, 23 cells from five male mice; cKO, 30 cells from nine male mice). (**I**) Representative images of biocytin-filled L2/3 (left) and L5 (middle) neurons, along with representative images of apical and basal dendritic segments (right). (**J**) Summary graphs for spine densities of apical (control, 13 cells from five male mice; cKO, 16 cells from nine male mice) and basal (control, 8 cells from three male mice; cKO, 16 cells from nine male mice) dendrites measured from L2/3 neurons. (**K**) Summary graphs for spine densities of apical (control, 22 cells from six male mice; cKO, 20 cells from nine male mice) and basal (control, 14 cells from five male mice; cKO, 25 cells from nine male mice) dendrites measured from L5 neurons. Two-tailed Mann-Whitney *U* test. Data are presented as means ± SEM.

On the basis of our findings of decreased OXPHOS in *Npas3*-deficient cortical astrocytes and lactate content in the PFC of *Npas3* cKO mice, we hypothesized that alterations in astrocyte metabolism would induce electrophysiological changes in neurons, leading to cortical circuit dysfunction. To test this hypothesis, we conducted whole-cell patch-clamp recordings in layer 2/3 (L2/3) and layer 5 pyramidal neurons (L5) in medial PFC (mPFC) of control or *Npas3* cKO mice (Fig. 4, A and D). There was no genotype-related difference in intrinsic excitability between control and *Npas3* cKO L2/3 pyramidal neurons (Fig. 4, B and C), as evidenced by compatible action potential frequencies in response to depolarizing current steps. In contrast, L5 neurons of *Npas3* cKO mice exhibited significantly decreased intrinsic excitability compared to L5 neurons of control mice (Fig. 4, E and F). The differences in excitability were observed at higher current injections, while the basal membrane properties (resting membrane potentials, input resistance, and rheobases) were not different between the control and *Npas3* cKO mice in either layer (Fig. 4, G and H). The significantly decreased intrinsic excitability (current-spike frequency relationship) in L5, but not in L2/3, neurons suggests that astrocyte-specific deletion of *Npas3* leads to decreased neuronal excitability and that astrocyte-neuron interactions could vary in a layer-specific manner.

### Astrocytic *Npas3* deficiency lowers spine density in pyramidal neurons

Astrocytes are known to be critically involved in synaptic formation, maturation, and maintenance among nearby neurons. Changes in electrophysiological functions in neurons may reflect homeostatic changes in synaptic inputs to the neuron or directly reflect synaptic dysfunction of the pathological conditions. Therefore, we next investigated whether *Npas3* deletion–mediated astrocyte dysfunction induced any alterations in the number of synapses onto cortical neurons. We included biocytin in the internal solution during whole-cell patch-clamp recordings to visualize the dendritic morphologies and spine distributions of the recorded neurons (Fig. 4I). We analyzed a total of 7492 spines on apical dendrites and 5766 spines on basal dendrites from 81 biocytin-filled neurons. We found no significant difference in the dendritic spine density along the apical dendrites of either L2/3 or L5 pyramidal neurons, but an analysis of the spine density along basal dendrites revealed significantly decreased dendritic spine density for both L2/3 and L5 pyramidal neurons of *Npas3* cKO mice compared to those of control mice (Fig. 4, J and K). Collectively, our electrophysiological and morphological studies demonstrate that reduced mitochondrial respiration in *Npas3*-deficient astrocytes leads to changes in the intrinsic excitability of pyramidal neurons in a layer-specific manner and decreased dendritic spines, which are critical for glutamatergic neurotransmission in the brain.

### Astrocytic *Npas3* deficiency leads to cognitive impairment, rescued by l-lactate injection

**Fig. 5.**
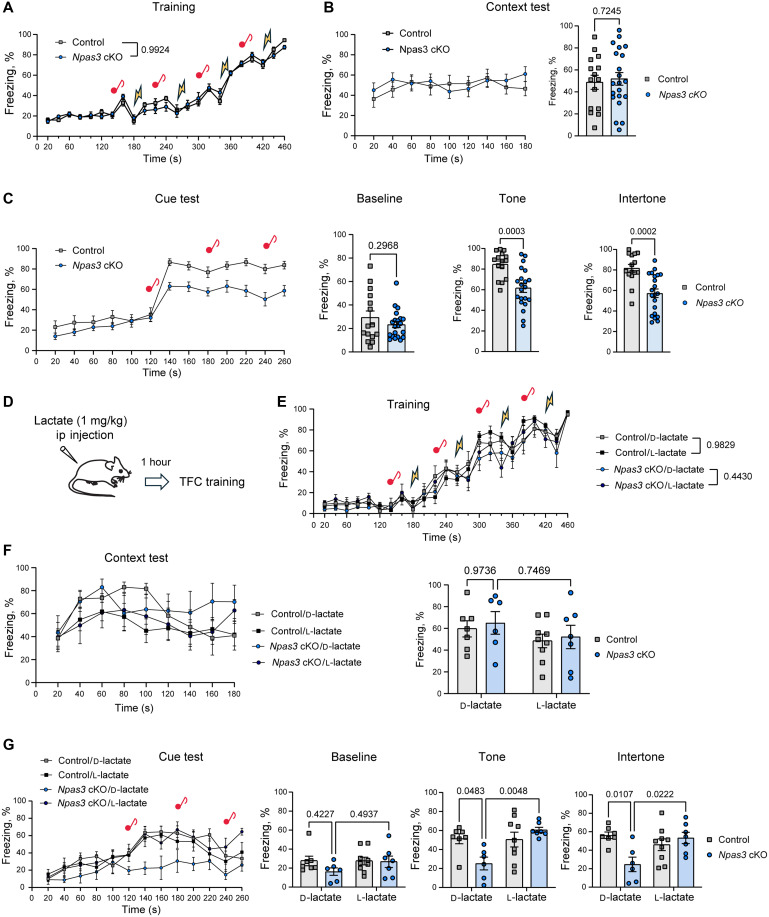
Astrocyte-specific *Npas3* cKO mice exhibit impaired TFC that is rescued by lactate. (**A**) Training session freezing; repeated-measures two-way ANOVA. (**B**) Context-dependent freezing over time and the average levels of context-dependent freezing. (**C**) Cue-dependent freezing was measured over time, before the first tone presentation, during tone presentation, and during the intertone intervals (control, nine male and six female mice; *Npas3* cKO, 11 male and 10 female mice; unpaired two-tailed Student’s *t* test). (**D**) Schematic of lactate injection [1 mg/kg, intraperitoneally (ip)] 1 hour before the training session. (**E**) Training session freezing following d- or l-lactate administration (repeated-measures two-way ANOVA). (**F**) Context-dependent freezing over time and the average levels of context-dependent freezing. (**G**) Cue-dependent freezing was measured over time, before the first tone presentation, during tone presentation, and during the intertone intervals (control/d-lactate, four male and three female mice; control/l-lactate, five male and four female mice; *Npas3* cKO/d-lactate, three male and three female mice; *Npas3* cKO/l-lactate, four male and three female mice; two-way ANOVA, followed by Tukey post hoc test). Data are presented as means ± SEM.

Given that deficient astrocyte bioenergetics, including altered mitochondrial OXPHOS, have been implicated in abnormal neuronal activity and/or synaptic transmission and memory impairment (*4*, *36*), we assessed the behavioral and cognitive phenotypes of *Npas3* cKO mice. We found no genotype-dependent differences between control and *Npas3* cKO mice in locomotor or rearing activity in the open field (OF) test and anxiety-like behaviors in the elevated plus maze test (fig. S5, A to C). No genotype-related differences were found in spatial alternation in Y maze, novel object, or place recognition tests (fig. S5, D to F). Therefore, we decided to run trace fear conditioning (TFC) test, a more challenging cognitive test (*37*), in which the unconditioned stimulus (foot shock) and the conditioned stimulus (tone) are separated by a time interval (*38*) (fig. S5G). We found no sex-dependent differences between control and *Npas3* cKO mice in TFC test (fig. S6, A to C); therefore, results from male and female mice were combined for the final analysis and presentation of the TFC results (Fig. 5). No group differences were found in freezing during the training session (Fig. 5A) and the context-dependent freezing (Fig. 5B). In contrast, *Npas3* cKO mice exhibited deficient cue-dependent fear memory as demonstrated by decreased freezing behavior during the tone presentation and the intertone intervals (Fig. 5C). These results indicate that *Npas3* cKO mice exhibit cognitive impairment as assessed by TFC. *Npas3* cKO mice showed similar freezing behavior at baseline (before the first tone presentation) in the cue test, suggesting comparable preexisting fear and activity levels to those in control mice (Fig. 5C). Overall, our study demonstrates that *Npas3* cKO mice exhibit attenuated cue-dependent freezing in a more challenging TFC test.

### Lactate treatment rescues impaired TFC in *Npas3* cKO mice

Prior reports have demonstrated the role of lactate produced by astrocytes in cognition (*39*–*41*). Thus, we sought to evaluate the effects of lactate treatment on the attenuated cue-dependent freezing in TFC in *Npas3* cKO mice. We found that a single intraperitoneal injection of l-lactate at a dose of 1 mg/kg, 1 hour before the TFC training session (Fig. 5D) increased cue-dependent freezing (both freezing during the tone and intertone) in *Npas3* cKO mice (Fig. 5G). In contrast, d-lactate, a stereoisomer control, had no effects in the TFC test. To assess potential confounds associated with the biological activity of d-lactate and its potential effects on mitochondrial metabolism (*42*), we compared the effects of d-lactate and saline on the intrinsic excitability of mPFC L5 neurons at either 1 or 24 hours after injection (fig. S7, A to F), as well as behavioral responses in the TFC test (fig. S7, G to I) of WT mice, and observed no treatment-related differences. Thus, systemic administration of l-lactate can rescue the cognitive abnormality associated with selective deletion of the *Npas3* gene in astrocytes.

### Astrocytic *Npas3* deficiency in the mPFC decreases excitatory synaptic neurotransmission and reduces spine density

**Fig. 6.**
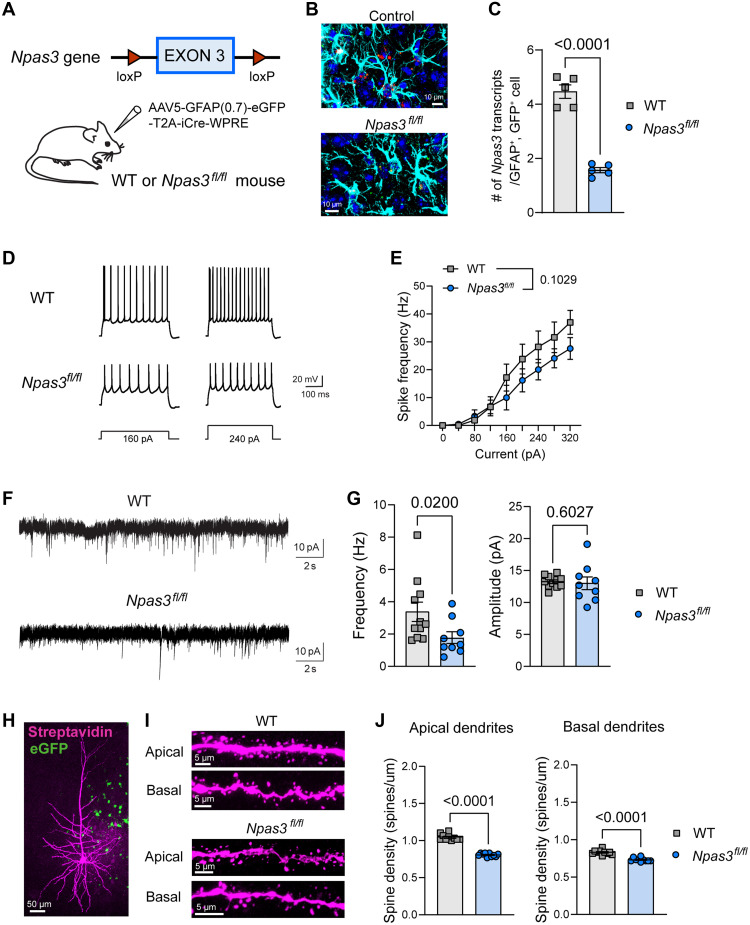
AAV-induced decrease in *Npas3* in mPFC astrocytes reduces excitatory synaptic transmission and spine density. (**A**) Strategy used to generate astrocyte-specific *Npas3* KD in mPFC. (**B**) Representative confocal images of dual BaseScope ISH for *Npas3* mRNA (red punctate dots), GFAP (cyan), and green fluorescent protein (GFP) (green) IF. Sections were counterstained with DAPI fluorescent dye to disclose tissue morphology, i.e., the cell nuclei (blue). All images were obtained using a 63× objective. (**C**) Quantitative analysis of *Npas3* mRNA expression in the mPFC after AAV-mediated *Npas3* KD. Data points represent individual animals. An average of 25 transduced astrocytes per mPFC were analyzed (*n* = 5 male mice per group; unpaired Student’s *t* test, two-tailed). (**D**) Representative voltage traces evoked by 160- and 240-pA current steps. (**E**) Current-spike frequency summary graph for L5 pyramidal neurons (WT, seven cells from four male mice; *Npas3^fl/fl^*, seven cells from four male mice; repeated-measures two-way ANOVA). (**F**) Representative current traces. (**G**) Summary graphs for mEPSC frequency and amplitude (WT, 11 cells from three male mice; *Npas3^fl/fl^*, 9 cells from three male mice; two-tailed Mann-Whitney *U* test). (**H**) Representative confocal images of biocytin-filled mPFC L5 pyramidal neurons and (**I**) representative images of apical and basal dendritic segments. (**J**) Summary graphs for spine densities of apical (WT, 10 cells from four male mice; *Npas3^fl/fl^*, 10 cells from seven male mice) and basal (WT, 10 cells from four male mice; *Npas3^fl/fl^*, 10 cells from seven male mice) dendrites measured from the neurons. Two-tailed Mann-Whitney *U* test. Data are presented as means ± SEM.

The *Npas3* cKO transgenic model does not allow for assessment of the regional effects of *Npas3* deletion. In addition, the GLAST promoter in the Cre line used has been shown to be active in both neurogenic astroglia–like cells and astrocytes throughout the brain, including the Bergmann glia of the cerebellum and the Müller glia in the retina (*34*, *35*, *43*, *44*). To address the above limitations of *Glast*-CreER^T2^–mediated recombination, we used an adeno-associated virus (AAV) [AAV5-GFAP(0.7)-EGFP-T2A-iCre-WPRE (AAV-Cre)] to knock down expression of *Npas3* in astrocytes of the PFC, the area we found electrophysiological and morphological abnormalities in pyramidal neurons (Fig. 4, A to O). We first validated the localization of viral vector injection in the mPFC (fig. S8A) and verified cell-specific AAV expression, confirming transduction of astrocytes, but not microglia or neurons (fig. S8B) in the mPFC. The efficiency of knockdown (KD) with this AAV was confirmed by an almost threefold decrease in *Npas3* mRNA expression in transduced glial fibrillary acidic protein (GFAP)–positive astrocytes (Fig. 6, B and C). We assessed the effects of astrocyte-specific *Npas3* KD on mPFC neurons and found a moderate decrease in excitability (*P* = 0.1029) in neurons in the vicinity of *Npas3* KD astrocytes (Fig. 6, D and E). We also investigated the effects of *Npas3* KD on synaptic functions and structure of the mPFC neurons and observed a significant reduction in the frequency of miniature excitatory postsynaptic currents (mEPSCs) (Fig. 6, F and G). Consistent with the mEPSC changes, dendritic spine density on mPFC L5 neurons of *Npas3^fl/fl^* mice was also reduced (Fig. 6, H to J). Together, these findings suggest that mPFC-specific astrocytic *Npas3* KD can alter neuronal structure and function.

### Astrocytic *Npas3* KD affects astrocyte morphology and induces alterations in synaptic markers

**Fig. 7.**
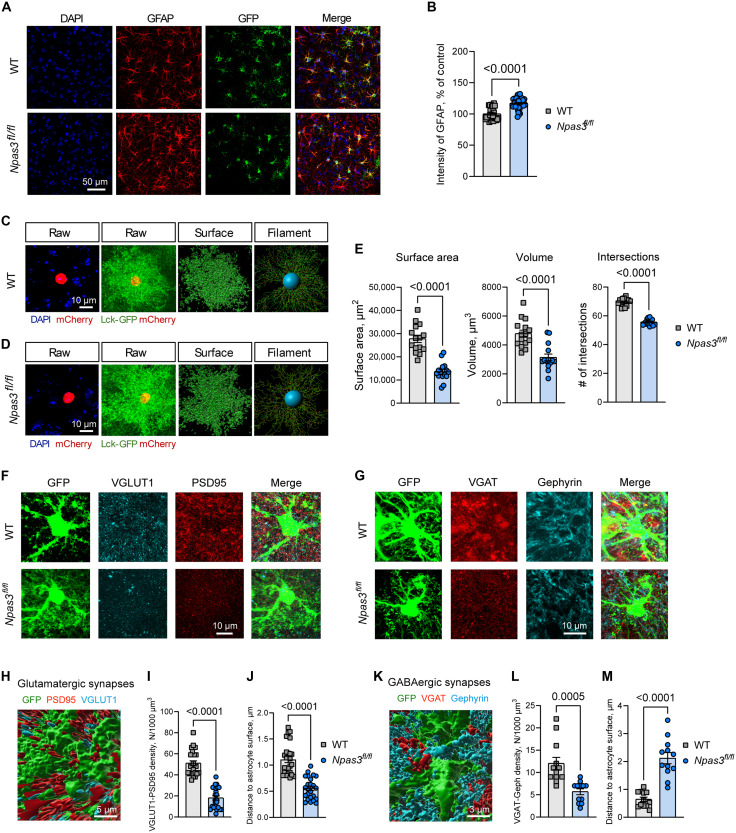
AAV-induced decrease in *Npas3* in mPFC astrocytes affects astrocyte morphology and synaptic markers. (**A**) Confocal images of GFP^+^ astrocytes (green) in the mPFC of WT or *Npas3^fl/fl^* mice, costained with GFAP (red) and DAPI (blue), 20× objective. (**B**) Quantification of GFAP^+^ fluorescence intensity in transduced (GFP^+^) astrocytes. GFAP^+^ fluorescence intensity was normalized to the WT values (WT, 30 cells from three male mice; *Npas3^fl/fl^*, 43 cells from four male mice). (**C** and **D**) Representative confocal and 3D-reconstructed images (Surface and Filament modules, Imaris) of WT and *Npas3^fl/fl^* astrocytes transduced with AAV5-GfaABC1D-Lck-GFP (green) to visualize astrocyte morphology, costained with DAPI (blue), 63× objective. AAV8-GFAP-mCherry-Cre (red) was used to induce astrocyte-specific recombination (*Npas3* KD). (**E**) Quantitative analysis of astrocyte surface area, volume, and Sholl intersections (WT, 15 cells from three male mice; *Npas3^fl/fl^*, 15 cells from three male mice). (**F**) Representative images of GFP^+^ astrocytes (green) costained with VGLUT1 (cyan) and PSD95 (red). (**G**) Representative images of GFP^+^ astrocytes (green) costained with VGAT (red) and Gephyrin^+^ (cyan). (**H**) 3D visualization of the costained area with VGLUT1^+^ (cyan) and PSD95 (red) puncta within an astrocyte zone. Summary graphs for (**I**) spatial density of VGLUT1^+^/PSD95^+^ puncta and (**J**) distance from VGLUT1^+^ puncta to the astrocyte surface (WT, 25 cells from five male mice; *Npas3^fl/fl^*, 24 cells from four male mice). (**K**) 3D visualization of the costained area with VGAT^+^ (red) and Gephyrin^+^ (cyan) within an astrocyte zone. Summary graphs for (**L**) spatial density of VGAT^+^/Gephyrin^+^ puncta and (**M**) distance from VGAT^+^ puncta to the astrocyte surface (WT, 12 cells from four male mice; *Npas3^fl/fl^*, 12 cells from four male mice). Images were obtained using a 63× objective (unpaired Student’s *t* test, two-tailed). Data are presented as means ± SEM.

To assess the cellular effects of *Npas3* KD on levels of the astrocyte marker GFAP and astrocyte morphology, we measured the intensity of GFAP in transduced [green fluorescent protein–positive (GFP^+^)] astrocytes and found significantly increased GFAP immunoreactivity (Fig. 7, A and B). To assess potential morphological abnormalities in *Npas3*-deficient astrocytes, we coexpressed AAV5-GfaABC1D-Lck-GFP and AAV8-GFAP-mCherry-Cre in WT or *Npas3^fl/fl^* mice to label astrocytes with and without *Npas3* KD (Fig. 7, C and D). Compared to WT astrocytes, we found significant morphological changes in *Npas3*-deficient astrocytes, including decreases in area, volume, and area/volume ratio, suggesting less ramified morphology, and increases in branch diameter (thicker processes) and simpler branching (Fig. 7E). Sholl analysis revealed fewer intersections, indicating less complex arborization in *Npas3*-deficient astrocytes (Fig. 7E).

Abnormal energy metabolism in astrocytes has been shown to affect excitatory and inhibitory synapses (*45*, *46*). Thus, to examine whether AAV-produced KD of *Npas3* affects cortical synapses, we measured the density of vesicular glutamate transporter 1 (VGLUT)1^+^/PSD95^+^ (glutamatergic synapses) and vesicular GABA transporter (VGAT)^+^/Gephyrin^+^ [γ-aminobutyric acid–releasing (GABAergic) synapses] located within individual territories of transduced astrocytes (Fig. 7, F to H and K). We found significantly reduced the density for both markers in *Npas3^fl/fl^* mice compared to WT mice. *Npas3* KD significantly reduced density of VGLUT1^+^/PSD95^+^ puncta located farther away from the surface of the transduced astrocyte (Fig. 7, I and J). Similarly, the density of VGAT^+^/Gephyrin^+^ puncta pairs was decreased in the mPFC of *Npas3^fl/fl^* mice compared to WT mice (Fig. 7L). In contrast, the distance from the surface of the transduced astrocytes was increased for VGAT^+^/Gephyrin^+^ puncta (Fig. 7M). Thus, KD of *Npas3* in PFC astrocytes altered expression of the pre- and postsynaptic markers of glutamatergic and GABAergic synapses within the individual territories of transduced astrocytes.

### Astrocytic *Npas3* KD elevates the expression of mitochondrial markers

**Fig. 8.**
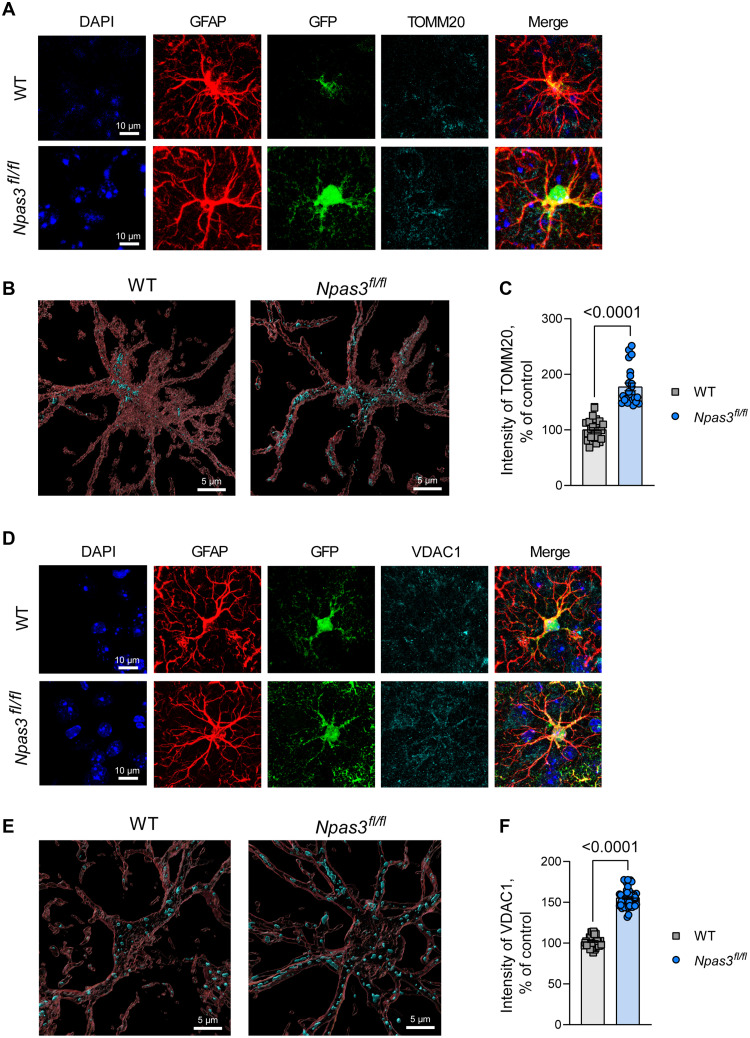
AAV-induced decrease in *Npas3 in* mPFC astrocytes elevates expression of mitochondrial markers. (**A**) Representative images of GFP^+^ astrocytes (green) costained with GFAP (red), TOMM20 (cyan), and DAPI (blue). (**B**) 3D-reconstructed images (Surface module) of astrocytes (GFAP, red) costained with TOMM20 (cyan). (**C**) Quantification of TOMM20 fluorescence intensity in transduced (GFP^+^) astrocytes. TOMM20 fluorescence intensity was normalized to values obtained from WT (control) samples (WT, 24 cells from four male mice; *Npas3^fl/fl^*, 23 cells from four male mice). (**D**) Representative images of GFP^+^ astrocytes (green) costained with GFAP (red), VDAC1 (cyan), and DAPI (blue). (**E**) 3D-reconstructed images (Surface module) of astrocytes (GFAP, red) costained with VDAC1 (cyan). (**F**) Quantification of VDAC1^+^ fluorescence intensity in transduced (GFP^+^) astrocytes. VDAC1 fluorescence intensity was normalized to values obtained from WT (control) samples (WT, 44 cells from four male mice; *Npas3^fl/fl^*, 53 cells from four male mice). All images were obtained using a 63× objective (unpaired Student’s *t* test, two-tailed). Data are presented as means ± SEM.

As *Npas3-*deficient astrocytes exhibit decreased OXPHOS (Figs. 2, A and B, and 3, F and G), we sought to determine whether *Npas3* deficiency affects mitochondrial biogenesis (*47*). We assessed mPFC expression of key mitochondrial protein-coding genes: translocase of the outer mitochondrial membrane 20 (TOMM20) and voltage-dependent anion channel 1 (VDAC1), which are involved in ATP production and maintenance of mitochondrial membrane integrity (*48*, *49*). Compared to WT mice, we found a significant increase in the expression of TOMM20 (Fig. 8, A to C) and VDAC1 (Fig. 8, D to F) in transduced (GFP^+^) astrocytes of *Npas3^fl/fl^* mice, suggesting increased mitochondrial content in *Npas3-*deficient mPFC astrocytes.

### *Npas3-*deficient mPFC astrocytes produce cognitive impairment that can be rescued by l-lactate injection

**Fig. 9.**
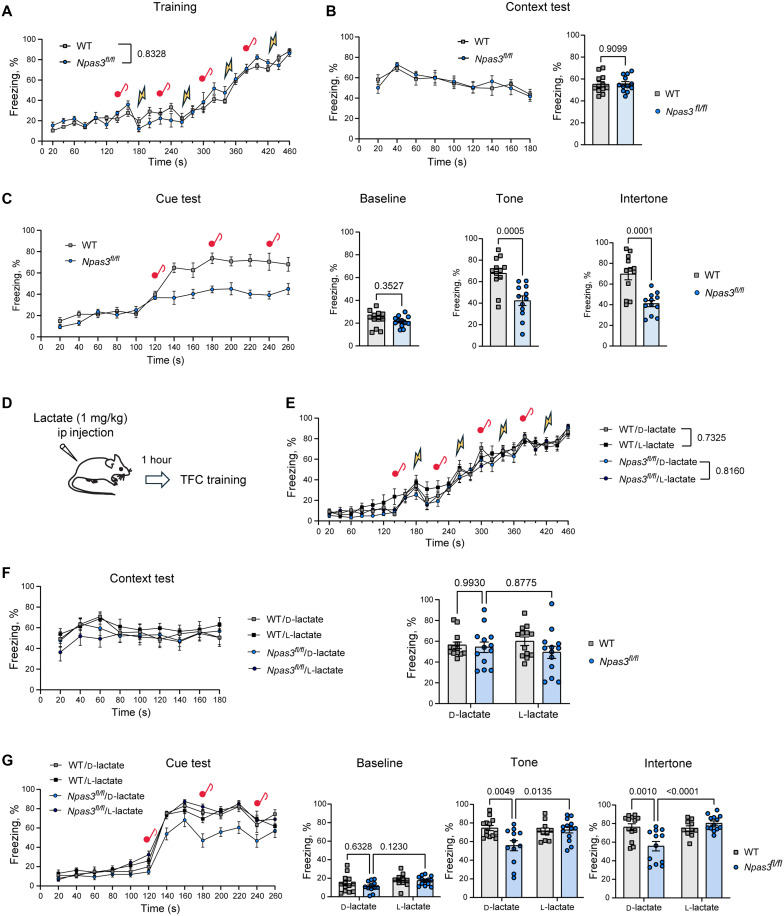
AAV-mediated *Npas3* KD in mPFC astrocytes impairs TFC, rescued by lactate. (**A**) Training session freezing (repeated-measures two-way ANOVA). (**B**) Context-dependent freezing over time and the average levels of context-dependent freezing. (**C**) Cue-dependent freezing was measured over time, before the first tone presentation, during tone presentation, and during the intertone intervals (WT, six male and six female mice; *Npas3^fl/fl^*, six male and six female mice; unpaired Student’s *t* test, two-tailed). (**D**) Schematic of lactate injection (1 mg/kg, ip) 1 hour before the training session. (**E**) Training session freezing following d- or l-lactate administration (repeated-measures two-way ANOVA). (**F**) Context-dependent freezing over time and the average levels of context-dependent freezing. (**G**) Cue-dependent freezing was measured over time, before the first tone presentation, during tone presentation, and during the intertone intervals (WT/d-lactate, three male and 10 female mice; WT/l-lactate, three male and seven female mice; *Npas3^fl/fl^*/d-lactate, three male and nine female mice; *Npas3^fl/fl^*/l-lactate, three male and 10 female mice; two-way ANOVA, followed by Tukey post hoc test). Data are presented as means ± SEM.

As we observed TFC impairment in *Npas3* cKO mice, we next investigated the effects of AAV-induced *Npas3* KD on TFC. As no sex-dependent differences were observed in the TFC test (fig. S9, A to C), the results from male and female mice were combined for analysis (Fig. 9). Consistent with the result observed in *Npas3* cKO mice, AAV-mediated KD of *Npas3* in PFC astrocytes did not alter freezing during the training session (Fig. 9A), nor did it affect context-dependent freezing (Fig. 9B). In contrast, AAV-mediated KD significantly attenuated cue-dependent freezing (Fig. 9C). We then examined whether the AAV-induced TFC deficit could be ameliorated by lactate. *Npas3^fl/fl^* and WT mice were injected with a single intraperitoneal injection of l-lactate at a dose of 1 mg/kg, 1 hour before the training session (Fig. 9D). l-Lactate, but not d-lactate, restored freezing levels during the tone presentation and the intertone intervals in *Npas3^fl/fl^* mice (Fig. 9G) without affecting freezing during the training or the context test (Fig. 9, E and F). Further, we found that intraperitoneal lactate treatment moderately increased excitability of neurons in the vicinity of *Npas3* KD astrocytes, as assessed 24 hours after the injection (fig. S10), suggesting that intraperitoneal l-lactate can increase neuronal excitability affected by *Npas3* KD in astrocytes.

## DISCUSSION

**Fig. 10.**
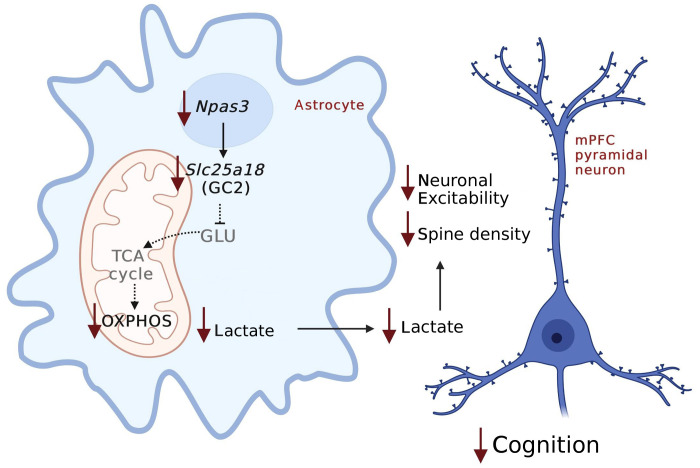
Schematic illustration of the mechanistic link between NPAS3-dependent astrocyte mitochondrial bioenergetics and cognitive function. Deletion of astrocytic *Npas3* leads to decreased expression of *Slc25a18* and its protein product, mitochondrial GC2, which transports glutamate (GLU) across the mitochondrial outer membrane. Glutamate can be oxidized in the TCA cycle. Diminished GC2 expression leads to decreased mitochondrial OXPHOS. Glutamate oxidation in astrocytes can be a source of lactate via the pyruvate pathway. Decreased secretion of lactate by *Npas3*-deficient astrocytes can contribute to reduced excitability of mPFC pyramidal neurons, decreased mEPSCs, and lower spine density in mPFC pyramidal neurons. These neuronal and synaptic changes can lead to cognitive impairment. Solid arrows indicate increase or decrease changes; dashed lines depict hypothesized outcomes. Created in BioRender, K. Murlanova (2026); https://BioRender.com/p52j453.

In this study, we present the findings that astrocytic NPAS3 is involved in astrocyte mitochondrial bioenergetics by regulating the expression of GC2 that mediates glutamate oxidation in mitochondria. We demonstrate that decreased levels of GC2 in astrocytes lead to decreased OXPHOS, reduced production and secretion of lactate by astrocytes, decreased excitability of PFC pyramidal neurons, and impaired cognitive function that can be rescued by lactate treatment (Fig. 10).

### Astrocytic NPAS3

NPAS3 is a transcription factor containing a bHLH PAS domain, predominantly expressed in the brain. It was recently identified as being disrupted by a genetic translocation in a family affected by schizophrenia (*11*). The human and mouse NPAS3 proteins share 90% sequence identity, suggesting that they play similar roles in the central nervous system (*50*). Mouse studies support the human findings, suggesting that NPAS3 may play a part in the neurobiology of neuropsychiatric conditions (*16*, *17*). As in the developing human fetal brain, NPAS3 is highly expressed in the ventricular zone (VZ) and the dentate gyrus of the hippocampus (*51*). Prior studies with *Npas3* KO or KD mice have focused on the VZ or hippocampal neurons. *Npas3* KO mice were shown to exhibit a reduced spine density in dentate gyrus granular neurons and cortical pyramidal neurons (*9*, *18*). In addition, *Npas3* KD in cortical VZ progenitor cells by in utero electroporation at embryonic day 14.5 (E14.5) led to impaired neuronal radial migration and disrupted cortical lamination (*52*). Behavioral assessments of *Npas3* constitutive KO mice revealed cognitive impairment, diminished social recognition, impaired prepulse inhibition, and hyperlocomotion (*9*, *16*, *17*).

Despite the name, recent research has identified that *Npas3* is highly enriched in astrocytes, and the *Npas3* gene exhibits higher expression in cortical astrocytes than in the whole cortex in mice (*9*). The *Npas3* gene is involved in the mechanisms of astrogenesis and astrocyte production. Constitutive *Npas3* KO mice exhibit impaired specification of radial glial cells into astrocytes, resulting in reduced astrocyte numbers in the developing cortex (*9*). KD of the gene in astrocytes during early development has been reported to reduce spine density on L2/3 pyramidal neurons and alter social behaviors, including reduced separation-induced ultrasonic vocalizations in neonatal mutant mice and decreased social novelty preference in adult mutant mice (*9*). However, the role of *Npas3* in adult astrocytes remains obscure. We provide the first evidence that *Npas3* is involved in the regulation of astrocyte mitochondrial bioenergetics in adult mice. Our findings expand prior data on the metabolic changes in *Npas3* KO mice (*10*) and point to a previously unidentified mechanism whereby reduced levels of NPAS3 in astrocytes can decrease mitochondrial respiration, leading to a reduction of lactate production and secretion, and cognitive dysfunction.

### GC2, glutamate oxidation, and lactate

We demonstrate that one of the mechanisms mediating the effects of *Npas3* deficiency in astrocytes on mitochondrial OXPHOS is related to reduced expression of GC2. Similar to GC1 encoded by *SLC25A22*, GC2 encoded by the *SLC25A18* gene works as a symporter and transports glutamate and a proton over the mitochondrial inner membrane (*53*, *54*). Inside the mitochondrial matrix, glutamate dehydrogenase converts glutamate into α-ketoglutarate (*55*–*57*) that is further metabolized in the TCA cycle (*58*, *59*). Both GC1 and GC2 are expressed in astrocytes (*60*), with GC2 being mostly present in astrocyte endfeet mitochondria (*30*, *31*). Prior studies suggested that levels of GC1 and GC2 in the brain could vary in a cell type–specific, brain region–, and age-related manner consistent with the idea of their functional heterogeneity (*28*), including distinct kinetic parameters (*61*).

More important for our study is the work of McKenna (*62*), which showed that glutamate is oxidized by astrocytes at a rate higher than that of glucose, 3-hydroxybutyrate, glutamine, lactate, or malate and that none of these substrates could effectively decrease the oxidative metabolism of glutamate. The complete oxidation of one glutamate molecule can yield approximately 20 molecules of ATP (*33*), and a lack of glutamate oxidation results in a lower global ATP level (*63*). Our results are consistent with the notion that suppressed glutamate oxidation in astrocytes reduces mitochondrial energy metabolism. We found that *Npas3*-related GC2 deficiency in astrocytes reduced astrocytic OXPHOS both in vitro and in vivo.

Diminished astrocytic OXPHOS was accompanied by decreased intracellular and extracellular lactate in astrocytes. It was proposed that in addition to glycolysis, lactate could also be a product of glutamate oxidation in astrocytes (*64*). It was shown that the carbon skeleton of glutamate enters the TCA cycle and can be completely oxidized via the TCA cycle and pyruvate recycling pathway (*65*). Using ^13^C-glutamate and ^13^C nuclear magnetic resonance spectroscopy in primary astrocytes, Sonnewald *et al.* (*66*) have reported that a greater amount of ^13^C was incorporated into lactate compared to glutamine. Consistent with this notion, our results show that GC2 overexpression restored lactate levels in *Npas3*-deficient astrocytes, supporting the hypothesis that oxidative metabolism of glutamate in astrocytes can contribute to lactate production by astrocytes (*67*).

Notably, in contrast to prior reports (*68*, *69*), *Npas3* deletion–induced decrease in OXPHOS was not accompanied by significant alterations in glycolysis, as assessed by ECAR and PER, as well as major glycolysis-related metabolites. It is possible that OXPHOS deficiency, coupled with limited changes in glycolysis, can lead to increased levels of glutamine incorporation and glutamine anaplerosis (*70*, *71*). With glutamine anaplerosis, cells compensate for OXPHOS deficiency not by boosting glycolysis but by increasing uptake and catabolism of glutamine to maintain the TCA cycle intermediates necessary for energy production and biosynthesis. While this needs to be studied more in depth, we hypothesize that glutamine anaplerosis may explain the complex interaction between glycolysis and OXPHOS in energy metabolism in our models.

There are other possible scenarios by which *Npas3* deficiency could alter astrocyte metabolism, leading to a simultaneous decrease in OXPHOS and lactate levels without substantially altering the overall glycolytic proton flux. For example, lactate production depends on the cytosolic redox state, which is mitochondria dependent. In astrocytes, a chronic OXPHOS deficit can lead to reduced pyruvate conversion to lactate if NAD^+^ regeneration is limited, resulting in impaired mitochondrial–cytosolic redox balance (*72*). In this scenario, the lactate pool decreases even if glycolytic flux (i.e., protons released) remains unchanged (as measured by ECAR and PER). Given that OXPHOS is a major route for NAD^+^ regeneration (*73*, *74*), this may represent an alternative mechanism.

Another possible pathway is shunting pyruvate into alternative pathways. Specifically, pyruvate produced by glycolysis may be diverted away from lactate production toward alanine (via transamination), TCA cycle intermediates (anaplerosis), and other biosynthetic pathways (*75*–*78*). As a result, levels of lactate can decrease, while glycolysis (H^+^ production) remains unchanged.

In addition, astrocytes’ consumption of lactate seems to depend on cell metabolic state. Lactate can be converted back to pyruvate by lactate dehydrogenase and funneled into the mitochondria and the TCA cycle (*79*). One could suggest that deleting *Npas3* in astrocytes might redirect the metabolic pathway of lactate, enhancing its oxidation as a compensatory mechanism for reduced OXPHOS. For example, several studies have confirmed that l-lactate may be directly oxidized to pyruvate in mitochondria through a “cytosol–mitochondrial lactate shuttle” (*80*–*84*). The involvement of lactate in the TCA cycle and the oxidative energy–generating machinery was also shown for astrocytes, namely, l-lactate can be taken up and metabolized by mitochondrial lactate dehydrogenase in human astrocytoma cells to produce ATP via OXPHOS (*79*). While the focus of the present work is to study the role of NPAS3 in glutamate oxidation in mitochondria, it will be critical in future studies to identify the specific molecular pathways that could explain the links between glutamate oxidation and lactate metabolism in our model.

### Lactate to support neuronal function and cognition

We found decreased intracellular and extracellular levels of lactate produced and secreted by *Npas3^+/−^* astrocytes. Our findings expand prior reports that decreased levels of extracellular lactate can lead to decreased neuronal excitability (*32*, *85*, *86*). Lactate is released by astrocytes and taken up by neurons, where it is oxidized in mitochondria to increase ATP levels. This leads to an increased [ATP]/[adenosine 5′-diphosphate] ratio and the closure of ATP-dependent potassium (K_ATP_) channels, promoting membrane depolarization and neuronal excitability (*32*, *85*, *86*). Thus, reduced neuronal excitability, decreased excitatory synaptic transmission, and decreased dendritic spines found in *Npas3* cKO and KD mice can be, at least in part, explained by the chronic shortage of extracellular lactate, which is required for maintaining a normal range of action potential firing.

Our electrophysiological data demonstrate that decreased expression of *Npas3* in astrocytes leads to decreased intrinsic excitability of pyramidal neurons in a layer-specific manner, namely, L5 pyramidal neurons exhibit a significantly decreased current-spike frequency relationship, whereas L2/3 pyramidal neurons do not.

Similar to our observation, recent studies have reported excitability changes selectively in L5 neurons, but not in L2/3 neurons, in the mPFC of adult mouse models of psychiatric disorders (*87*, *88*). This layer-specific vulnerability may be partly attributable to differences in cellular composition between L2/3 and L5. Although both layers contain intracortical neurons and projection neurons targeting the nucleus accumbens and the basolateral amygdala, L5 specifically comprises corticothalamic neurons projecting to the mediodorsal thalamus in the mPFC. As the principal cortical output layer enriched in large, long-range projecting pyramidal neurons, L5 is likely to impose greater metabolic demands, including increased reliance on lactate utilization. Consequently, L5 neurons exhibit impaired excitability in the decreased lactate level, since lactate is an energy substrate for cortical neurons and known to enhance the neuronal firing activity (*89*).

Further, consistent with a prior study (*9*), we found reduced spine density on basal, but not apical, dendrites of pyramidal neurons in both L2/3 and L5 pyramidal neurons in the *Npas3* cKO mice and reduced spine density on both apical and basal dendrites in L5 pyramidal neurons in the *Npas3^fl/fl^* mice (PFC-*Npas3* KD). These findings suggest that additional mechanisms may contribute to the layer-specific neuronal changes in electrophysiological and structural properties in response to astrocyte-specific *Npas3* deletion. For example, pyramidal neurons (*90*–*92*) and interneurons (*93*–*95*) are known to be layer-specifically different in the cortex, thus contributing to layer-specific changes in physiological functions under certain pathological conditions.

In addition, astrocytes themselves are cortical layer-specifically different (*96*–*98*). A previous report categorized cortical astrocytes into upper-layer (L2 to L4) and lower-layer (L5 to L6) astrocytes by presenting highly enriched gene sets specific to each layer (*96*). Notably, astrocytes in the upper and lower cortical layers display distinct morphologies, characterized predominantly by radial and tangential elongation, respectively. Moreover, upper-layer astrocytes occupy a larger territorial volume and exhibit more extensive ensheathment of synaptic clefts compared with their lower-layer counterparts. These tighter astrocyte–axodendritic interactions in the upper layers may render the spike excitability of L2/3 neurons, as well as the apical dendrites of both L2/3 and L5 neurons, less susceptible to dysfunction arising from astrocytic *Npas3* deficiency. Thus, depending on their localization, astrocytes may use different mechanisms and regulate different properties of adjacent neurons. Together, altered neuronal excitability and mEPSCs, along with changes in dendritic spine density in pyramidal neurons, demonstrate impaired synaptic neurotransmission in the cortical synaptic network, which may link the abnormal metabolism of *Npas3*-deficient astrocytes to the cognitive dysfunction observed in the mice.

It was previously proposed that astrocyte bioenergetics play a role in on-demand energy support, e.g., during difficult cognitive tasks (*4*). In line with this, *Npas3* cKO mice did not show significant alterations in basic behavioral and cognitive tests but exhibited impairment in a more challenging cognitive test, namely, TFC. TFC has been found to place a greater demand on the proper interaction between the frontal cortex and hippocampus (*37*, *99*, *100*), the brain regions with a considerable reduction of *Npas3* expression in cKO mice. This finding was further corroborated and refined using AAV-mediated KD of *Npas3* expression in PFC astrocytes. The impaired freezing during the cue test was rescued by l-lactate administration in both mouse models (*Npas3* cKO and *Npas3^fl/fl^* mice). l-Lactate was administered before learning because astrocyte-derived lactate is known to support neuronal activity and synaptic plasticity during memory encoding and consolidation (*40*, *101*–*103*), processes that occur at or shortly after training. Thus, restoring lactate availability at the time when memory traces are formed can prevent the downstream memory deficits observed in mice with astrocytic *Npas3* deficiency. The AAV-produced cognitive phenotype was associated with decreased levels of glutamatergic synaptic markers within the individual territories of affected astrocytes, suggesting underlying neuronal dysfunction.

*Npas3*-deficient mPFC astrocytes exhibited elevated GFAP expression and pronounced morphological alterations, indicating that NPAS3 is critical for maintaining astrocytic cellular homeostasis (*104*). This notion is consistent with prior reports that decreased OXPHOS makes astrocytes more vulnerable to metabolic stress, leading to the development of astrocytic reactivity (*105*–*107*). In addition to elevated levels of GFAP, *Npas3*-deficient astrocytes exhibited higher expression of TOMM20 and VDAC1, the mitochondrial markers. The up-regulation of mitochondrial markers may reflect a compensatory response to impaired mitochondrial bioenergetics. Alternatively, mitochondrial fragmentation contributes to the elevated mitochondrial markers: Fission generates smaller, more numerous mitochondria, which can be reflected in increased TOMM20^+^ and VDAC1^+^ immunoreactivity without improving bioenergetic efficiency. Fragmented mitochondria are associated with impaired electron transport and ATP production (*108*, *109*), consistent with the reduced OXPHOS observed in *Npas3*-deficient astrocytes.

Our study has some limitations. The metabolic consequences of *Npas3* deletion were mainly studied in astrocytes of the frontal cortex. However, the role of *Npas3* in astrocytes of different brain regions may vary. Further, identification of a neuronal circuit underlying TFC and the effects of systemic lactate may help explain some inconsistencies in the effects of lactate on the mPFC electrophysiological measures between cKO mice and mice injected with AAV. We focus our study on *Slc25a18*, but, as our RNA-seq data and those from other laboratories have demonstrated, NPAS3 regulates the expression of multiple genes involved in energy metabolism and other cellular processes in astrocytes that can modulate neuronal functions (*9*, *10*). Although we provide evidence for the association between GC2 and lactate, future studies will need to determine the underlying metabolic mechanisms in much greater detail.

Together, our study reveals mechanistic insights regarding how the deficiency of an important transcriptional factor in astrocytes impairs astrocyte energy metabolism, neuronal function, and complex cognitive behavior. Our findings provide detailed biochemical mechanisms for the astro-neurotransmitter interactions responsible for neuronal and behavioral alterations while highlighting the mPFC as a pivotal brain region in *Npas3*-mediated pathophysiology.

## MATERIALS AND METHODS

### Mice

The animal study protocols were approved by the Institutional Animal Care and Use Committees of the University at Buffalo (number 202000034, approved on 20 July 2020), Johns Hopkins University (number MO18M01, approved on 24 January 2018), and Korea Brain Research Institute (number 25-00035, approved on 20 August 2025). Animal experimentation was conducted in accordance with the US National Research Council’s Guide for the Care and Use of Laboratory Animals and the US Public Health Service’s Policy on Humane Care and Use of Laboratory Animals.

Mice were given standard laboratory chow and water ad libitum and were housed in a temperature-controlled (21° ± 2°C), humidity-controlled (55 ± 5%), and light-controlled (reversed 12-hour light/12-hour dark cycle, light on at 6:00 p.m. and light off at 6:00 a.m.) room.

#### Constitutive KO of Npas3

Heterozygous *Npas3^+/−^* (HET) KO mice (*16*) [a gift from A. Pieper (University of Iowa); strain #031112, the Jackson Laboratory] were used for RNA-seq (E16 to E17) and primary cultures of astrocytes [postnatal day 1 (P1)]. *Npas3^+/−^* were on the C57BL/6J background and were bred to *Npas3^+/−^* (HET) or C57BL/6J (WT). *Npas3^+/+^* littermates served as control (WT) mice.

#### Conditional astrocyte-specific KO of Npas3

Mice with *Npas3* cKO were generated by crossing GLAST-CreER mice [a gift from J. Nathans [Johns Hopkins University School of Medicine]; Tg(Slc1a3-cre/ERT)1Nat/J; strain #012586, the Jackson Laboratory] with *Npas3^fl/fl^* mice [a gift from A. Pieper (University of Iowa)]. GLAST is highly expressed in adult astrocytes, and knock-in of CreER^T2^ into the GLAST locus leads to tamoxifen-sensitive recombination in all astrocytes with endogenous GLAST promoter activity (*34*). Male and female mice (GLAST::CreER^T2^ × *Npas3fl^/+^*) were the experimental group. Cre-negative male and female littermates carrying the floxed *Npas3* allele (*Npas3^fl/+^*) obtained from the same GLAST::CreER^T2^ × *Npas3^fl/fl^* breeding were used as controls. All mice were on the C57BL/6J background. Mice were reared by their dams until P21; afterward, four same-sex mice were housed per cage. At P30, *Npas3* cKO and control mice were injected intraperitoneally for three consecutive days with tamoxifen (100 mg/kg; T5648, MilliporeSigma) dissolved in sunflower seed oil (S5007, MilliporeSigma), which induced conditional recombination in astrocytes of *Npas3* cKO mice. Mice used for the electrophysiology experiment were injected with the same dose of tamoxifen for three consecutive days at P21 to P26. The dose volume was 10 ml/kg of body weight.

*Npas3* cKO and their corresponding control mice were used for behavioral tests, electrophysiological recordings, morphological measurements, metabolic flux, and biochemical assays as well as rescue (lactate) experiments.

#### AAV-mediated Npas3 KD

To KO *Npas3* expression in PFC astrocytes, AAV5-GFAP(0.7)-EGFP-T2A-iCre-WPRE (1.2 × 10^13^ genome copies/ml; VB4888, Vector Biolabs) was injected into the prelimbic area of the mPFC [anterior-posterior (AP), +2.0; medial-lateral (ML) ±0.3; dorsal-ventral (DV), −2.0]. This AAV expresses EGFP-T2A-iCre driven by an astrocyte GFAP(0.7) promoter, and the AAV5 serotype has shown to have the highest astrocytic specificity (*110*). AAV vector was delivered bilaterally in the target region in the amount of 250 nl at the rate of 1 nl/s to *Npas3^fl/fl^* and control (WT) mice (P46) under the isoflurane anesthesia as previously described (*111*). Two weeks after recovery from stereotactic surgery and maximal virus expression, adult (P60) *Npas3^fl/fl^* and control mice were used for the experiments.

### Animal genotyping

Mouse genotypes from tail biopsies were determined using real-time polymerase chain reaction (PCR) with specific probes designed for each gene (Transnetyx, Cordova, TN).

### RNA-seq and analysis

Forebrain tissue was harvested from *Npas3^+/+^* (WT; *n* = 5) and *Npas3^+/−^* (HET; *n* = 4) female mice at E16 to E17. Total RNA was extracted using the miRNeasy Micro Kit (217084, QIAGEN), subjected to polyadenylate capture, and followed by TruSeq library preparation. Paired-end sequencing was performed on an Illumina HiSeq 2000; however, only single-end reads are used in downstream analyses due to technical failure in sequencing that affected secondary reads. Reads were aligned to the mouse reference genome GRCm38.p6 using the STAR aligner (version 2.7.9a) (*112*) and annotated with GENCODE release M25. Transcripts were gene level quantified with featureCounts from the Subread package (version 2.0.0) (*113*). Normalization and differential expression analysis with shrinkage of effect size for log fold change estimates were completed with DESeq2 in the R computing environment (*114*). Multiple test correction was completed with the Benjamini-Hochberg method, and all differentially expressed genes reported herein are below a FDR-adjusted *P* value of 0.05 (FDR < 0.05). Differential expression rank order for GSEA was performed using the fgsea package (*115*) in R and applied to the Hallmark, M2 (CP), M3 (regulatory target), and M5 (ontology: gene ontology biological process) gene sets from the Mouse MSigDB (*116*). Metabolic pathways from Kyoto Encyclopedia of Genes and Genomes (KEGG) and Pathway Studio were assessed with the R package, MetaPhOR, that uses a bootstrapping approach to determining statistical significance of transcriptionally inferred dysregulation of metabolic pathways (*117*).

### Primary cultures of astrocytes

Astrocytes in primary cultures were obtained from the cortex of 1-day old *Npas3^+/−^* (HET) and *Npas3^+/+^* (WT) mouse neonates. Cell suspensions were seeded in T75 culture flasks in Dulbecco’s modified Eagle’s medium (DMEM) with glucose (4.5 g/liter) and l-glutamine (11965092, Life Technologies) supplemented with 10% fetal bovine serum (FBS; 10437028, Life Technologies), 1 mM sodium pyruvate (11360-070, Life Technologies), and 0.1% penicillin/streptomycin (120-095-721, Quality Biological) and incubated at 37°C in a humidified 5% CO_2_-containing atmosphere. To detach nonastrocytic cells, after 7 days in vitro (DIV), the flasks were shaken at 180 rpm for 30 min on an orbital shaker to remove microglia and at 240 rpm for 6 hours to remove oligodendrocyte precursor cells (*118*). The supernatant was discarded, and the attached, astrocyte-enriched cells were reseeded in the appropriate for the assay plates. Cells were used at 9 DIV.

### Cell transfections

For *Slc25a18* (GC2 protein) overexpression experiments, we used *Slc25a18* (Myc-DDK–tagged) expression plasmid (MR218451, OriGene) or an empty vector (pCMV6-Entry; PS100001, OriGene). Transfections were performed with Lipofectamine 2000 Reagent (11668027, Thermo Fisher Scientific) according to the manufacturer’s protocol. Cells were used after 24 to 48 hours.

### Immunomagnetic isolation of astrocytes from adult brain tissue

Brain astrocytes were isolated at P90 from *Npas3* cKO (GLAST::CreERT2 × *Npas3^fl/+^*) and control mice (for Western blotting and the metabolic flux assay). Dissected brain tissue (PFC) was placed in Dulbecco’s phosphate-buffered saline (PBS) containing Ca^2+^, Mg^2+^, d-glucose, and pyruvate (14287080, Thermo Fisher Scientific) and fragmented with a sterile scalpel. Astrocytes were isolated according to the “Isolation and cultivation of astrocytes from adult mouse brain” Miltenyi Biotec protocol for the Adult Brain Dissociation kit (130-107-677, Miltenyi Biotec, Bergisch Gladbach, Germany) and anti–ACSA-2 MicroBead kit (130-097-678, Miltenyi Biotec). After magnetic sorting, cells were centrifuged for 5 min at 300*g* at 4°C. Pellets were dissolved in cell lysis buffer (9803, Cell Signaling Technology, Danvers, MA, USA) for Western blotting or in AstroMACS medium (130-117-031, Miltenyi Biotec) for subsequent cultivation. We confirmed the identity of the isolated astrocytic fraction by Western blotting against astrocytic (S100β), neuronal nuclei protein (NeuN), and microglial (Iba1)–specific markers. Mouse primary cultures of astrocytes, neurons, and microglia prepared as previously described (*41*, *119*, *120*) served as positive controls for the cell type–specific markers.

### Real-time PCR analyses

Total RNA was isolated using the PureLink RNA Mini Kit (12183025, Invitrogen) according to the manufacturer’s instructions and quantified by spectrophotometric analysis. An aliquot of each RNA sample was then treated with deoxyribonuclease (18068015, Invitrogen) to avoid DNA contamination. Quantitative real-time PCR was performed on the CFX384 Touch Real-Time PCR Detection System using the iTaq Universal SYBR Green One-Step Kit (1725151, Bio-Rad) and 5 ng of RNA per reaction. The primers were (forward and reverse, respectively) 5′-AGCTCGACAAGGCATCCATC-3′ and 5′-GCCTTCGTTGTGCACCTTTT-3′ for *Npas3*; 5′-GCTCTGACGTACGTGGTCTC-3′ and 5′-TTGGGGCTTCCGAACATCTC-3′ for *Slc25a18*; 5′-CGGCATGTATAGGGGAGCAG-3′ and 5′-AGGTCAGCTTCTGTCCATCC-3′ for *Slc25a22*; and 5′-GGCAAGTTCAACGGCACAGTCAAG-3′ and 5′-GCACATACTCAGCACCAGCATCAC-3′ for glyceraldehyde-3-phosphate dehydrogenase (*Gapdh*). The samples were run in 384-well formats, and four replicate reactions were performed for each sample. PCR reaction conditions are as follows: 50°C for 10 min; 95°C for 3 min; 40 cycles of 95°C for 10 s; and 60°C for 30 s, including a dissociation curve at the last step to verify single amplicon in the reaction. The mRNA abundance of each transcript was normalized to that of *Gapdh* obtained in the same sample. Relative target gene expression was calculated according to the 2(−ΔΔ*C*_T_) method (*121*). The resulting normalized values in *Npas3^+/−^* primary astrocytes were expressed as the fold change versus the corresponding normalized values in control (*Npas3^+/+^*) astrocytes.

### Protein determinations

Protein samples were quantified by the BCA Protein Assay Kit (23227, Thermo Fisher Scientific) using bovine serum albumin as a standard.

### Western blotting

Cells (primary astrocytes, neurons, microglia, or immunomagnetically isolated astrocytes) were sonicated for 30 s in cell lysis buffer (9803, Cell Signaling Technology) containing 1 mM phenylmethylsulfonyl fluoride (10837091001/PMSF-RO, MilliporeSigma). Cell lysates were spun down at 10,000*g* for 10 min at 4°C. The resulting supernatant was processed by SDS–polyacrylamide gel electrophoresis, and the separated proteins were transferred onto a nitrocellulose membrane. The membrane was washed in tris-buffered saline solution containing 0.05% Tween 20 (TBST) and was blocked for 1 hour at room temperature (RT) in TBST containing 5% nonfat dry milk (9999, Cell Signaling Technology). The membrane was incubated overnight at 4°C with rabbit anti-GC-1 (1:1000; ab137614, Abcam), rabbit anti- SLC25A18 (1:500; 17348-1-AP, Proteintech), mouse anti-NeuN (1:200; MAB377, MilliporeSigma), mouse anti-Iba1 (1:500; MA5-27726, Invitrogen) antibodies, or OXPHOS mouse antibody cocktail (1:1000; 45-8099, Invitrogen), followed by corresponding horseradish peroxidase–conjugated anti-rabbit (1:1000; NA934, Cytiva) or anti-mouse (1:1000, NA931, Cytiva) secondary antibodies. The immunoblots were visualized on Blu-Lite autoradiography films (A8813, MTC Bio) using Super Signal West Pico Chemiluminescent Substrate (34080, Thermo Fisher Scientific). The optical density of protein bands on each digitized image was normalized to the optical density of β-actin (A5441, MilliporeSigma) or S100β (ab52642, Abcam) band using ImageJ software (version 1.49v).

### RNA ISH for *Npas3*

ISH was performed using the “BaseScope” method combined with immunofluorescence (IF) (*122*, *123*) and 15-μm fixed-frozen sagittal mouse brain sections obtained from *Npas3* cKO (*GLAST*::CreER^T2^ × *Npas3^fl/+^*), *Npas3^fl/fl^* after AAV-5-GFAP(0.7)-EGFP-T2A-iCre–mediated astrocyte-restricted KD of *Npas3* in the prelimbic area of the mPFC and their respective control mice (P90). ISH-IF was performed with BaseScope Detection Reagent Kit v2-RED (323910, ACD), a custom “3ZZ” probe for *Npas3* mRNA (1186601-C1, ACD; GenBank accession NM_013780.3; nucleotides 432 to 620) and chicken anti-GFAP (1:100; Aves Labs) or rabbit anti-GFP primary antibodies (1:20; G10362, Thermo Fisher Scientific) with the corresponding Alexa Fluor 488– or Alexa Fluor 633–labeled species-specific secondary antibodies (1:1000; A11039, A32731, and A21103, Life Technologies of Thermo Fisher Scientific) according to the manufacturer’s instructions (Technical Note MK 51-149/Rev. B, ACD) with a following modification: Protease III (322337, ACD) was used for the brain tissue pretreatment during the integrated codetection workflow. Nuclei were counterstained with 4′,6-diamidino-2-phenylindole (DAPI; 0.5 μg/ml; D1306, Life Technologies of Thermo Fisher Scientific), and slides were mounted in VECTASHIELD antifade medium (H-1000, Vector Laboratories). Images were obtained on an inverted confocal microscope Leica-Sp8 using LASX software. Consistent laser settings were used for all imaging sessions: 405-, 488-, and 568-nm lasers.

Confocal z-stacks of ISH-IF tissues were acquired using a 63× oil objective, with a 1-μm step size. Images for each mouse were collected from at least two sections of tissue. A total of 16 optical planes were collected in z-stacks and collapsed for image analysis. Images were processed using Imaris 10.1.0 software (Oxford Instruments). The BaseScope Fast Red signal is fluorescent in the red channel, and the GFAP in the green channel. Individual *Npas3* mRNA puncta were counted in each astrocyte using a custom Imaris macro in which astrocytes were determined on the basis of GFAP green signal (Imaris Surface module) and *Npas3* mRNA molecules were detected as red punctate dots (Imaris Spots module).

### Metabolic flux assays

The mitochondrial OCR was assessed in primary and immunomagnetically isolated astrocytes using a Seahorse XF24 Extracellular Flux Analyzer (Seahorse Bioscience) or a Seahorse XFe96 Extracellular Flux Analyzer (Agilent) with Seahorse Wave Desktop software (v.2.6.4). The OCR assay uses probes loaded on a sensor cartridge for fluorometric detection of extracellular medium O_2_ flux changes of cells. Astrocytes were plated at a density of 100,000 per well in XF24 cell-culture microplates or 50,000 per well in XFe96 cell culture microplates precoated with poly-d-lysine (P6407, MilliporeSigma) and incubated in AstroMACS cell (130-117-031, Miltenyi Biotec) medium for 24 hours. Then, the medium was replaced with Seahorse XF DMEM (103575-100, Agilent) supplemented with 10 mM glucose (G8769, MilliporeSigma), 1 mM l-glutamine (25030081, Life Technologies), and 1 mM sodium pyruvate (11360070, Life Technologies) and incubated at 37°C without CO_2_ for 45 min to allow cells to preequilibrate with the assay medium. OCR was determined at baseline and at each point after adding oligomycin [inhibits ATP synthase (complex V)], FCCP (uncoupling agent that collapses the proton gradient and disrupts the mitochondrial membrane potential), and rotenone (which inhibits complex I). Oligomycin (75351, MilliporeSigma), FCCP (C2920, MilliporeSigma), and rotenone (557368, MilliporeSigma), diluted in assay medium, were loaded into port A, port B, and port C, respectively. Final concentrations in cell culture microplates were 1 μM oligomycin, 1 μM FCCP, and 1 μM rotenone. OCR was measured after each injection to determine mitochondrial or nonmitochondrial contribution to OCR as previously described (*36*). All measurements were in picomoles per minute and were normalized to either the total protein concentration (Seahorse XF24) or the cell number (Seahorse XFe96) in each well. Each sample was measured in triplicates. Experiments were repeated three times in biologically independent culture preparations.

During the mitochondrial stress test performed on the Seahorse XFe96 analyzer, PER was simultaneously calculated from extracellular acidification measurements using Seahorse Wave software and expressed as picomoles of H^+^ per minute per 1000 cells. Basal PER was determined from measurements obtained before oligomycin injection, and compensatory PER was calculated following oligomycin treatment to assess glycolysis-associated proton efflux under conditions of inhibited mitochondrial ATP synthesis.

Nonmitochondrial OCR was determined by OCR after injection of rotenone. Basal respiration was determined by starting level of cellular OCR, subtracting the nonmitochondrial OCR. Maximal respiration was determined by the maximum OCR rate after FCCP injection minus nonmitochondrial OCR. ATP production was determined by the last OCR measurement before oligomycin injection minus the minimum OCR measurement after oligomycin injection.

For the measurement of ECAR, culture medium in primary and immunomagnetically isolated astrocyte cultures plated at a density of 100,000 per well in XF24 cell culture microplates was replaced with Seahorse XF base medium (103334-100, Agilent) supplemented with 2 mM glutamine (103579-100, Agilent). Glycolytic flux, as assessed by ECAR, was analyzed by the sequential addition of glucose, oligomycin, and 2-DG (which inhibits glycolysis by competitively binding to glucose hexokinase) in an XF24 flux analyzer, as previously described (*41*). Glucose, oligomycin, and 2-DG (D8375, MilliporeSigma), diluted in assay medium, were loaded into port A, port B, and port C, respectively. Final concentrations in XF24 cell culture microplates were 10 mM glucose, 1 μM oligomycin, and 50 mM 2-DG. ECAR was measured after each injection to determine glycolysis, glycolytic capacity, and glycolytic reserve. Glycolysis is defined as the glucose-induced increase in ECAR and is calculated by subtracting nonglycolytic acidification (baseline measurement) from the highest ECAR measurement following the addition of glucose. Maximum glycolytic capacity was calculated as the difference between the highest ECAR measurement during nonglycolytic acidification and the highest ECAR measurement after the addition of oligomycin. Glycolytic reserve was calculated as the difference between ECAR after glucose and after oligomycin. ECAR values were in milli-pH per minute and normalized relative to the protein concentration in each well.

### Lactate assay

Lactate was measured in cell pellets (intracellular lactate), cell culture medium (extracellular lactate), brain tissues, CSF, and blood serum using a Lactate-Glo assay kit (J5021, Promega). Cells were seeded at a density of 20,000 cells per well in 96-well plates precoated with poly-d-lysine and incubated in DMEM (11965092, Life Technologies) supplemented with 10% FBS (10437028, Life Technologies) and 0.1% penicillin/streptomycin (120-095-721, Quality Biological) at 37°C in a humidified incubator with 5% CO_2_ for 24 hours. Then, the medium was replaced with FBS-free DMEM. After 3 hours of incubation, the medium was collected, centrifuged at 10,000*g* for 5 min, and supernatant was frozen at 20°C until further use. After the cell medium was removed, cells were washed with 0.01 M PBS (pH 7.4; 46-013-CM, Corning) to avoid contamination from lactate in the media, and inactivation solution (0.6 N HCl; 258148, MilliporeSigma) was added directly to the cells in the 96-well plate. After neutralization with 1 M tris base (Trizma; T1503, MilliporeSigma), samples were stored at −20°C until the assay.

CSF (5 to 7 μl) was collected from anesthetized mice by inserting a glass capillary into cisterna magna as previously described (*124*), and the collected CSF was centrifuged at 10,000*g* for 10 min at 4°C to remove any tissue debris and stored at −20°C until the assay. Mouse serum was obtained from the tail vein (*125*) and stored at 20°C until the assay. PFC was dissected and kept at −80°C until the assay. Cell culture medium, cell pellets, brain tissues, CSF, and blood serum were processed for lactate measurements according to the technical manual for J5022 (Promega). Luminescence was recorded by the BioTek Cytation 1 plate reader (Agilent). Samples of cell lysates and tissue homogenates were used for protein determination, and all measures were normalized to total protein concentration.

### In vitro [U-^13^C] glucose metabolite labeling

For the tracer experiment, astrocytes were seeded as quadruplicates into 35-mm tissue culture dishes and maintained in maintenance media consisting of advanced DMEM (12491015, Thermo Fisher Scientific) supplemented with 10% FBS, 4% sodium pyruvate, and 1% geneticin (10131035, Thermo Fisher Scientific). At roughly 80% confluency, the maintenance medium was removed, and the cells were washed three times with sterile 0.01 M PBS. Tracer medium consisting of glucose-free DMEM (A14430-01, Thermo Fisher Scientific) supplemented with 10 mM [U-^13^C] glucose (CLM-1396, Cambridge Isotope Laboratories), 10% dialyzed FBS (A3382001, Thermo Fisher Scientific), and 0.4% geneticin was then added. Cells were incubated at 5% CO_2_ and 37°C for 24 hours to reach isotopic steady state (*126*). After 24 hours, the medium was removed, and the plates were washed three times on ice with ice-cold 0.01 M PBS. One milliliter of ice cold 75% ethanol was added, and the cells were scraped using a rubber-tipped cell scraper, followed by centrifugation at 1200 rpm. Ethanol:0.1 M PBS (85:15) was added to the cell pellet at 25 μl of buffer per 10^6^ cells to quench cellular metabolism and initiate cellular lysis. Cellular lysates were collected in cryovials, snap frozen on dry ice, and stored at −80°C until mass spectrometry analysis. Samples were prepared and analyzed in the Roswell Park Comprehensive Cancer Center Bioanalytics, Metabolomics and Pharmacokinetics Shared Resource. For phosphate-containing metabolites, 10 μl of cell pellet supernatant was combined with 40 μl of 85% methanol. Samples were derivatized by adding 20 μl of *N*-(*t*-butyldimethylsilyl)-*N*-methyltrifluoroacetamide. Seventy microliters of water was added to the samples before additional shaking for 5 min at 1500 rpm. Samples were centrifuged, and the bottom layer was transferred to a plate for injection on the liquid chromatography–mass spectrometry (LC-MS) system. For amino acids, 5 μl of cell pellet supernatant was combined with 10 μl of water. Samples were derivatized by adding 50 μl of dansyl chloride (15 mg/ml) in acetone and 50 μl of 2 M sodium carbonate in water and incubating at 60°C, while shaking. A liquid/liquid extraction was performed by adding 400 μl of 3% acetic acid and 1 ml of ethyl acetate. The ethyl acetate layer was transferred and dried under a stream of nitrogen gas and reconstituted in 50 μl of 75% methanol. The plate was then injected into the LC-MS system. Samples were analyzed with an Agilent 1290 Infinity II LC system coupled to an Agilent 6545B quadrupole orthogonal acceleration–time-of-flight mass spectrometer. LC-MS data were processed using Agilent MassHunter Quantitative Analysis software, version 11.0.

### Behavioral tests

Behavioral tests were performed at P60 (4 weeks posttamoxifen or 2 weeks post–AAV injections). *Npas3* cKO and control mice were exposed to the OF, elevated plus maze, and TFC tests to assess locomotor activity, exploratory behavior, anxiety, and learning and memory, respectively. TFC in response to the acute effects of lactate was assessed in a separate cohort of *Npas3* cKO and control mice. *Npas3^fl/fl^* mice after AAV-mediated astrocyte-specific *Npas3* KO in the prelimbic cortex and control mice were exposed to the TFC only.

Mice were gently handled in the housing room for 1 min over the course of 3 to 5 days before behavioral testing. Mice were transported from the vivarium into the test rooms and were allowed to habituate for at least 30 min before testing. Mice were randomly and evenly allocated to each experimental group. To perform the group allocation in a blinded manner during data collection, animal preparation and experiments were performed by different investigators. All the experiments were carried out during the dark phase, and the red-light intensity was adjusted to ∼20 lux during the behavioral testing. The interval between different behavioral tests was at least 1 week. The tests were performed in the following order: OF test, elevated plus maze, and fear conditioning.

#### OF test

Activity in the OF was monitored and calculated using the Photobeam Activity System (San Diego Instruments). The Photobeam Activity System consists of an OF clear plastic chamber (50 cm by 50 cm) with photocell emitters and receptors equally spaced along the perimeter of the chamber (16 × 16 photobeam configuration). The photocell emitters and receptors create an *x*-*y* grid of invisible infrared beams. At the beginning of the test, each mouse was placed in the corner of the chamber. The animal’s movement led to beam breaks, which were calculated for 30 min. The number of beam breaks broken [horizontal as a measure of locomotion and vertical as a measure of exploratory behavior (i.e., rearing behavior)] was recorded and analyzed using Photobeam Activity System software (San Diego Instruments).

#### Elevated plus maze

Anxiety-related behavior was assessed via an elevated plus maze. Mice were placed in the center of the maze, consisting of two open arms and two closed arms, 33 cm in length (San Diego Instruments Inc., San Diego, CA, USA), and allowed to explore the maze for 5 min. The elevated plus maze was raised 0.5 m above the floor. ANY-maze Tracking software (v.6.34, Stoelting, Co.) was used to automatically record and analyze preference to the open arms of the maze (percentage of time spent in the open arms of the maze). The criterion for arm entries was the front two paws and the snout inside the arm.

#### Y-maze

The short-term spatial memory was assessed using the Y-maze. Spontaneous alternation behavior in the Y-maze was calculated using ANY-maze Tracking software. An alternation was defined as entries into all three arms on consecutive occasions. The number of maximum alternations equaled the total number of arm entries minus two, and the percentage alternation was calculated as (actual alternations/ maximum alternations) × 100.

#### Novel object and novel place recognition tests

The spatial and recognition memory were tested using the novel object recognition test (NORT) and novel place recognition test (NPRT) in the OF square arenas, tracked using the Top Scan tracking software (CleverSys Inc., VA, USA). The NORT was divided into three sessions: habituation, training, and test. During the habituation session, mice spent 10 min in the OF arena to reduce exploration of the novel environment and anxiety during the training and test sessions. During the training session, mice were habituated to two identical objects placed at fixed locations in the arena. Each mouse began the training session at the arena’s center, and the time spent in each object’s immediate vicinity (within 2 cm) was recorded during a 10-min period, after which the mouse was placed back into the home cage. To test short-term memory, each mouse was returned to the same arena 30 min after the training, with one of the two objects replaced by a new one (test session). The time spent near each object was recorded for 5 min. In the NPRT, the number of sessions and objects was the same as described for NORT, with the following adaptation: A mouse was presented with two similar objects during the training session, and then one of the object locations was changed in the testing session. The preference (%) for a novel object (or an object in a novel location) was calculated from the time the mice spent near each object by dividing the novel object (or object in a novel location) exploration time by the total time spent by the mouse near novel and familiar objects (or novel and familiar locations of the objects).

#### Trace fear conditioning

TFC was a 3-day test consisting of the day of habituation, the day of training, and the test day. Mice were habituated to the shock box (Coulbourn) for 10 min. The following day, mice were placed in the shock box, and four 90-dB tones were delivered for 20 s (at 140, 220, 300, and 380 s after the test started). Twenty seconds after the termination of the tone (at 180, 260, 340, and 420 s after the test started), a 0.5-mA shock was delivered for 2 s. The entire training protocol consisted of four repeated tone-shock pairings. The fear response, measured as the duration of freezing events in response to the tone-shock pairings, was assessed by Freeze Scan software (CleverSys Inc.). The percentage of freezing times was compared between mouse groups by a two-way repeated-measures analysis of variance (ANOVA) test using twenty-three 20-s intervals. On the third day, mice were placed in the shock box for 3 min, and the freezing behavior was assessed as a measure of contextual memory. Immediately following the context test, cue-dependent fear memory was evaluated. Mice were placed into a novel testing chamber for 4 min and 20 s. After a 120-s baseline period, the conditioned tone (90 dB, 20 s) was presented three times at 120, 180, and 240 s, without foot shock. Freezing behavior was analyzed during the baseline period, during tone presentations, and during the intertone trace intervals.

### Lactate administration

Sodium d-lactate (71716, MilliporeSigma) or sodium l-lactate (71718, MilliporeSigma), dissolved in normal saline, were injected intraperitoneally into mice at a dose of 1 mg/kg, 1 hour before the TFC training session, or 1 or 24 hours before brain slice electrophysiological recording. For all injections, the dose volume was 10 ml/kg of body weight.

### Immunostaining

Mice were deeply anesthetized with Fluriso (isoflurane, USP; VetOne, MWI Veterinary Supply Co.), followed by transcardial perfusion with ice-cold 0.01 M PBS and then 4% paraformaldehyde in 0.01 M PBS (sc-281692, Chem Cruz). The brains were dissected out and postfixed in 4% paraformaldehyde in 0.01 M PBS for 24 hours at 4°C. After cryoprotection in 30% sucrose (S5-500, Thermo Fisher Scientific) in 0.01 M PBS for 48 hours, the brains were embedded in OCT compound (4585, Thermo Fisher Scientific) and cut into 40-μm-thick coronal sections using a cryostat.

Transduced brain areas were visualized using expression of GFP. To test cell specificity of transduction, brain sections were stained with chicken anti-GFAP (1:1000; GFAP, Aves Labs), rabbit anti-Iba1 (1:1000; 019-19741, Wako Chemicals), or guinea pig anti-NeuN (1:1000; MAB377, MilliporeSigma) antibodies as previously described (*127*).

To evaluate coexpression of pre- and postsynaptic markers of GABAergic or glutamatergic synapses, guinea pig anti-VGAT (1:1000; 131004, Synaptic Systems) and mouse anti-Gephyrin (1:1000; 147021, Synaptic Systems) (for GABAergic synapses) or mouse anti-PSD95 (1:1000; P246, MilliporeSigma) and rabbit anti-VGLUT1 (1:1000; 48-2400, Thermo Fisher Scientific) (for glutamatergic synapses) antibodies were used as previously described (*128*). To evaluate the expression of mitochondrial markers, rabbit anti-TOMM20 (1:200; 11802-1-AP, Proteintech) and mouse anti-VDAC1 (1:1000; 14734, Abcam) were used. For VDAC1 immunostaining, antigen retrieval was performed as previously described (*129*), followed by a regular immunostaining protocol. Sections were rinsed with 0.01 M PBS and blocked with 3% normal goat serum (16210072, Thermo Fisher Scientific) and 0.3% Triton X-100 (11332481001, Roche Diagnostics) in 0.01 M PBS for 1 hour at RT, followed by incubation with the primary antibodies at 4°C overnight and with the corresponding Alexa Fluor 568– and Alexa Fluor 633–labeled species-specific fluorescent secondary antibodies (1:1000; A11041, A11011, A11075, A21052, A11005, and A21070, Thermo Fisher Scientific) for 2 hours at RT. After three rounds of 5-min 0.01 M PBS washes, sections were dried for 30 min and mounted on Superfrost Plus slides (12-550-15, Thermo Fisher Scientific) with VECTASHIELD Antifade Mounting Medium with DAPI (H-1200, Vector Laboratories) and stored at 4°C until imaging.

Images were collected with a Leica SP8 confocal microscope with photomultiplier tube and hybrid detector at the University at Buffalo Confocal Microscope and Flow Cytometry Facility. Laser settings and optical parameters were kept constant between all images in control (WT) and *Npas3^fl/fl^* groups.

Confocal images were acquired using a Plan-Apochromat 63×/1.40 Oil DIC (differential interference contrast) M27 objective at a zoom factor of 2.4×, with the astrocyte soma located at the center of the image and processes extending to fill the entire field of view (frame size, 1024 pixels by 1024 pixels; voxel size, 55 nm by 55 nm by 300 nm in *x*-*y*-*z*), yielding 70 to 90 optical sections per z-stack. Additional confocal image stacks were acquired using a 20×/0.75 CS2 objective at a zoom factor of 1.0 (frame size, 1024 pixels by 1024 pixels; voxel size, 55 nm by 55 nm by 500 nm in *x*-*y*-*z*), yielding 27 to 30 optical sections per z-stack.

### Analysis of synaptic markers

Single-cell Imaris-based (10.2.0; Oxford Instruments) analysis of the spatial density of the pre- and postsynaptic markers of excitatory and inhibitory synapses was performed within the zones of transduced GFP^+^ astrocytes. PSD95^+^ or VGLUT1^+^ immunoreactivity was used to evaluate glutamatergic synaptic puncta, while VGAT^+^/Gephyrin^+^ immunoreactivity was used to evaluate the GABAergic synaptic puncta. The total number of PSD95^+^/VGLUT1^+^ and VGAT^+^/Gephyrin^+^ pairs of puncta within zone of an astrocyte/GFP^+^ 3D cell surface (density, *N* per 1000 μm^3^), and the distance between synaptic puncta and the astrocyte surface were calculated as previously described (*128*), with the minor modifications, including smoothing with surface area detail level at 0.1 μm and local contrast with a diameter of the largest sphere of 0.5 μm and the threshold of 10 for both pre- and postsynaptic markers.

### Analysis of astrocyte morphology

To visualize and quantitatively analyze astrocyte morphology, AAV5-GfaABC1D-Lck-GFP (7 × 10^12^ genome copies/ml; 105598-AAV5, Addgene) was injected into the prelimbic area of the mPFC (AP, +2.0; ML, ±0.3; DV, −2.0) to label astrocyte membranes as previously described (*130*). *Npas3* expression in PFC astrocytes was knocked out as previously described (see the “AAV-mediated *Npas3* KD” section), using AAV8-GFAP-mCherry-Cre (4.1 × 10^12^ genome copies/ml; UNC Vector Core). A mixture of AAV vectors was delivered bilaterally into the target region at a volume of 250 nl per vector per side at a rate of 1 nl/s in *Npas3^fl/fl^* and control (WT) mice (P46). Four weeks after recovery from stereotactic surgery, mice were used for astrocyte morphology analysis. After tissue processing as previously described, confocal z-stack images of individual mCherry/Lck-GFP–expressing astrocytes were acquired. The z-stacks were imported into Imaris software, and three-dimensional (3D) reconstructions were generated. A “surface” was first built on the basis of the membrane-targeted Lck-GFP signal, allowing representation of the entire astrocyte, including fine peripheral processes; background was subtracted uniformly. The surface function within Imaris software was used to extract surface area and volume information for each cell. Subsequently, the Imaris Filament Tracer module was applied using consistent intensity-threshold parameters across all cells to semiautomatically or automatically trace and skeletonize astrocyte processes, as previously described (*128*, *131*). Quantitative morphometric parameters (e.g., volume/surface area, number of branch points, number of Sholl intersections, etc.) were extracted from the reconstructed skeleton and surface.

### Analysis of mitochondrial markers

Confocal z-stack images were acquired from brain sections containing transduced GFP^+^ astrocytes immunostained with anti-GFAP and either anti-TOMM20 or anti-VDAC1. Image stacks were imported into Imaris software for 3D reconstruction and fluorescence quantification. Astrocytes were segmented using the Surface module based on the GFAP and GFP signals, applying uniform background subtraction and threshold parameters across all samples. The resulting 3D surface object delineated the full astrocyte volume and was used to generate a masked channel containing only the mitochondrial marker fluorescence (TOMM20 or VDAC1) within the astrocyte boundary. Mean fluorescence intensity and volume-normalized intensity for each marker were extracted directly from the Imaris Surface Statistics output. All imaging and analysis parameters were kept constant across experimental groups. This workflow follows previously established methods using viral astrocyte labeling, confocal imaging, and Imaris surface-based quantification of immunofluorescent markers in astrocytes as previously described (*132*).

### Brain slice preparation and cell identification

*Npas3* cKO male mice (P34 to P59) were anesthetized with isoflurane, decapitated, and the brains were rapidly removed and chilled in ice-cold sucrose solution containing 76 mM NaCl, 25 mM NaHCO_3_, 25 mM glucose, 75 mM sucrose, 2.5 mM KCl, 1.25 mM NaH_2_PO_4_, 0.5 mM CaCl_2_, and 7 mM MgSO_4_ (pH 7.3). Acute brain slices (300 μm), including the mPFC, were prepared using a vibratome (VT-1200s, Leica), as previously described (*133*–*136*). Slices were then incubated in warm (32° to 35°C) sucrose solution for 30 min; then transferred to warm (32° to 34°C) artificial CSF (aCSF) composed of 125 mM NaCl, 26 mM NaHCO_3_, 2.5 mM KCl, 1.25 mM NaH_2_PO_4_, 1 mM MgSO_4_, 20 mM glucose, 2 mM CaCl_2_, 0.4 mM ascorbic acid, 2 mM pyruvic acid, and 4 mM l-(+)-lactic acid (pH 7.3), 315 mOsm; and allowed to cool to RT. All solutions were continuously bubbled with 95% O_2_/5% CO_2_. For whole-cell recordings, slices were transferred to a submersion chamber on an upright microscope [Zeiss Axio Examiner, objectives: 5×, 0.16 numerical aperture (NA), and 40×, 1.0 NA) fitted for infrared DIC microscopy. Slices were continuously superfused (2 to 4 ml/min) with warm oxygenated aCSF (32° to 34°C). Neurons were visualized with a digital camera (Sensicam QE, Cooke) using transmitted light. The mPFC was identified on the basis of the shape and location of the corpus callosum, and L2/3 and L5 were determined on the basis of the distance from the cell-sparse layer 1 and the large cell bodies of L5 neurons. Neurons having pyramidal cell bodies with prominent apical dendrites projecting toward layer 1 were targeted. Any neurons exhibiting firing patterns of fast-spiking interneurons were excluded from recordings.

### Whole-cell patch-clamp recordings

For the intrinsic excitability measurement and morphology analysis, glass recording electrodes (2 to 4 MΩ) were filled with an internal solution containing 2.7 mM KCl, 120 mM KMeSO_4_, 9 mM Hepes, 0.18 mM EGTA, 4 mM MgATP, 0.3 mM Na guanosine 5′-triphosphate, and 20 mM phosphocreatine (Na) (pH 7.3), 295 mOsm. Biocytin (0.25%, w/v) was added to the internal solution for filling cells during whole-cell recordings. Whole-cell recordings were performed in the presence of the following blockers of glutamate and γ-aminobutyric acid receptors: 5 μM 2,3-dioxo-6-nitro-7-sulfamoylbenzo[*f*]quinoxaline (AMPA receptor antagonist), 5 μM carboxypiperazin-4-yl-propyl-1-phosphonic acid (*N*-methyl-d-aspartate receptor antagonist), and 10 μM 6-imino-3-(4-methoxyphenyl)-1(6*H*)-pyridazinebutanoic acid hydrobromide [SR95531; γ-aminobutyric acid type A (GABA_A_) receptor antagonist; all from Tocris]. The resting membrane potential was measured after the whole-cell configuration was achieved. Neurons exhibiting a resting membrane potential greater than −60 mV were excluded from recordings. Whole-cell patch-clamp recordings were obtained using a Multiclamp 700B amplifier (Molecular Devices) and digitized using an ITC-18 (Instrutech) controlled by software written in Igor Pro (WaveMetrics). The series resistance was not compensated (initial series resistance was <30 MΩ for all recordings). The input resistance was determined by measuring the voltage change in response to a 1-s-long, −100-pA hyperpolarizing current step. The current-spike frequency relationship was measured with a range of depolarizing current steps presented in a pseudorandom order (1-s-long, 0 to 400 pA, 40-pA increments, 5-s interstimulus intervals). For each current intensity, the total number of action potentials exceeding 0 mV generated during each step was measured and then averaged across the three trials. The rheobase was determined by first probing the response of the neuron with 1-s-long depolarizing steps to define a small range of current steps that bounded the rheobase. The response of the neurons was then tested within this range using 1-s-long depolarizing steps with 1-pA increments. Neurons were held at −70 mV for the current-spike frequency relationship and rheobase measurements. Action potential properties (threshold, amplitude, and half-width) were measured from single spikes evoked by rheobase current injections. For the action potential threshold analysis, the *dV*/*dT* of the membrane potentials was measured, and the threshold was defined as the first time point at which the *dV*/*dT* exceeded 10. Action potential amplitude was measured from the action potential threshold to the peak voltage. Action potential half-width was measured at half the amplitude between threshold and the peak voltage. mEPSCs were recorded in the presence of picrotoxin (100 μM) to block GABA_A_ receptors and tetrodotoxin (1 μM), a blocker of voltage-dependent sodium current. L5 pyramidal neuron input and access resistance (10 to 20 MΩ) were continuously monitored throughout the experiment using 5-mV hyperpolarizing voltage steps (500-ms duration). Recordings were discarded when the input and series resistance changed by more than 20%. Membrane voltage and current were amplified using Axoclamp 2B, digitized with Digidata 1550B, and acquired using the pClamp 10.2 software (Molecular Devices, Union City, CA, USA). mEPSCs were analyzed with MiniAnalysis software (Synaptosoft, Decatur, GA). The synaptic events were selected using amplitude threshold (5 pA), rise time (1 ms), and area threshold (30 fC). All selected events were further visually inspected to prevent noise from compromising the analysis. All signals were low-pass filtered at 10 kHz and sampled at 20 to 100 kHz.

### Dendritic spine assay

To increase the efficiency of biocytin infusion, depolarizing current steps (10 nA at 100 Hz for 100 ms) were injected into the recorded neuron at the end of each cell recording (*133*). The recorded slices were fixed in 4% paraformaldehyde in 0.01 M PBS overnight. After rinsing with 0.01 M PBS, slices were incubated in 0.01 M PBS blocking solution containing 2% Triton X-100 (MilliporeSigma) for 1 hour at RT. To visualize biocytin-filled cells, slices were next incubated with 1% Triton X-100 and Alexa Fluor 647– or Alexa Fluor 555–conjugated streptavidin (S32357 or S32355, Thermo Fisher Scientific) overnight at 4°C. The following day, slices were rinsed with 0.01 M PBS. After rinsing, slices were mounted with Aqua-Poly/Mount (18606-20, Polysciences). Fluorescence images were taken with a confocal microscope (LSM 800, Zeiss; 63× objective lens) as z-stack (2-μm interval) tiled images to cover the extent of the cell’s dendritic processes. We measured the dendritic spine density using Neurolucida (MBF Bioscience). Spines were counted along 20- to 80-μm-long dendritic segments, located at least 50 μm away from the soma for apical dendrites and at least 30 μm away from the soma for basal dendrites. Three different dendritic segments were chosen per each dendrite, and the spine counts from each dendrite were averaged for individual neurons.

### Data analysis and statistical tests

Data analysis was performed in Igor Pro (WaveMetrics), Excel (Microsoft), and GraphPad Prism 10 software, except for RNA-seq analysis. Statistical significance was determined by two-tailed Student’s *t* test or two-tailed Mann-Whitney *U* test for simple comparisons and two-way ANOVA with post hoc Tukey test for multiple comparisons and interaction effects with or without corrections for repeated measures. Data distributions were tested with the Shapiro-Wilk normality test. Depending on the normality test results, (parametric) unpaired *t* test or (nonparametric) Mann-Whitney *U* test was used for comparing two groups. The test types and the results for each comparison are described in the figure legends. The Bonferroni correction was used for multiple behavioral tests on the same mice. Power analysis was conducted using G*Power 3.1.9.6 (*137*) based on previously published works (*4*, *127*) to determine the sample size. The experimenter was blinded to the mouse information during the experiments and raw data analysis. Significance was set at a *P* value of <0.05, and data are presented as the means ± SEM on the graphs.
