# Supplementary material for: NPAS3-regulated astrocyte mitochondrial bioenergetics is required for cognition
Source: Sci Adv. 2026 Jun 17;12(25):eadt2527. doi: 10.1126/sciadv.adt2527 (PMC13274620; doi:10.1126/sciadv.adt2527)
Supplement: Supplementary file 1 — Figs. S1 to S10 Legends for data S1 and S2 [file sciadv.adt2527_sm.pdf]

Science Advances
AAAS

Supplementary Materials for

NPAS3-regulated astrocyte mitochondrial bioenergetics is required for cognition

Kateryna Murlanova *et al.*

Corresponding author: Juhyun Kim, juhyun.kim@kbri.re.kr; Mikhail V. Pletnikov, mvpletni@buffalo.edu

Sci. Adv. **12**, eadt2527 (2026)
DOI: 10.1126/sciadv.adt2527

The PDF file includes:

Figs. S1 to S10
Legends for data S1 and S2

Other Supplementary Material for this manuscript includes the following:

Data S1 and S2

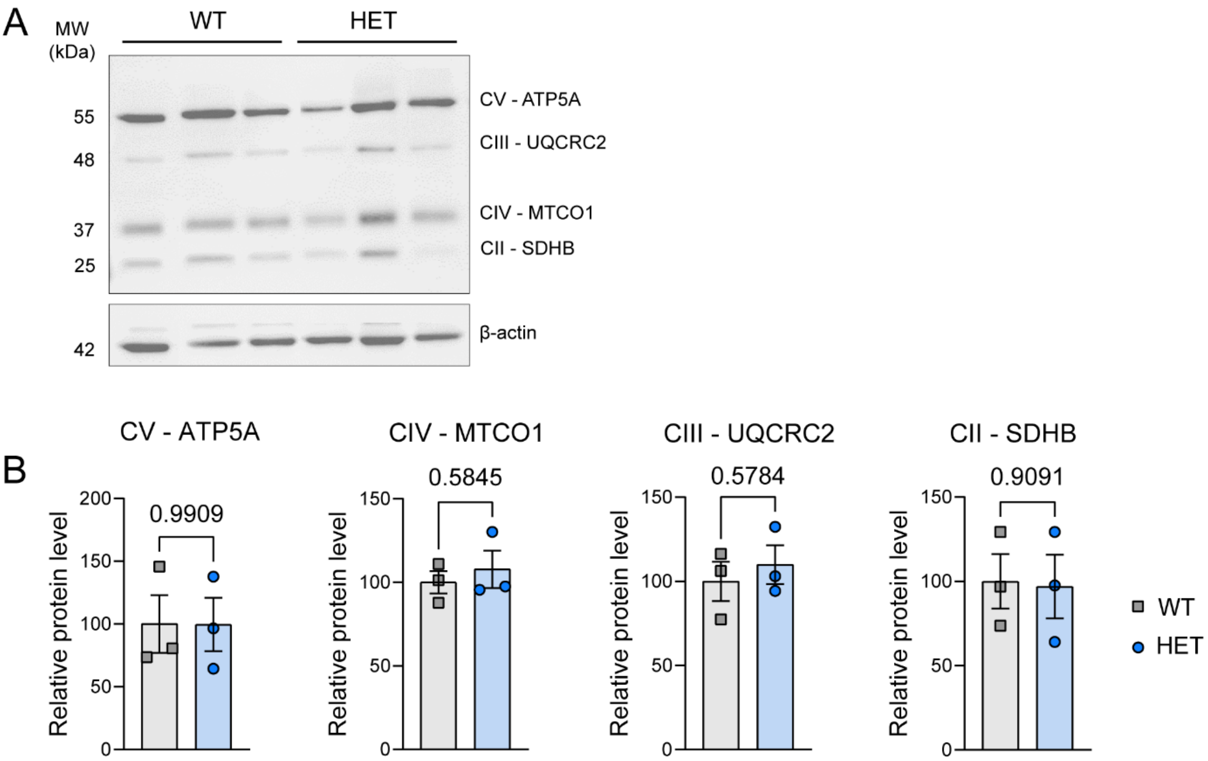

Fig. S1. No group difference in expression of OXPHOS complexes. (**A**) Representative immunoblots for the complexes V (ATP5A), IV (MTCO1), III (UQCRC2) and II (SDHB) in WT and *Npas3*$^{+/-}$ (HET) astrocytes. (**B**) Relative protein concentration of OXPHOS complexes normalized by abundance of β-actin [n = 3 biologically independent cell culture preparations; unpaired Student's *t*-test, two-tailed]. Data are presented as means ± SEM.

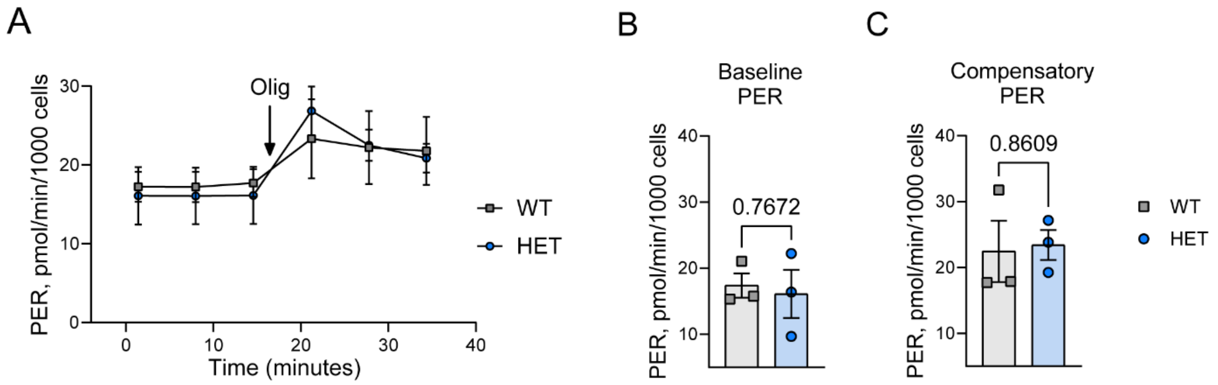

Fig. S2. No difference in glycolysis-associated proton efflux between WT and *Npas3*-deficient astrocytes. (**A**) PER for WT and *Npas3*$^{+/-}$ (HET) cortical astrocytes in primary culture. (**B**) Basal PER and (**C**) compensatory PER, following oligomycin (Olig) injection in WT and *Npas3*$^{+/-}$ (HET) astrocytes [n = 3 biologically independent cell culture preparations; unpaired two-tailed Student's *t*-test]. Data are presented as means ± SEM.

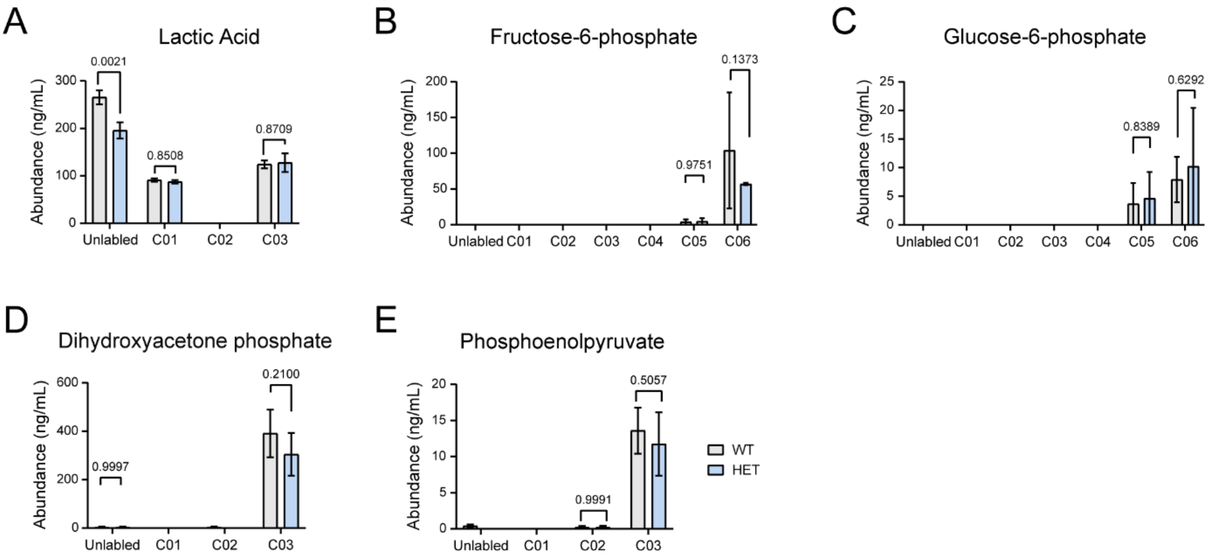

Fig. S3. Carbon labeling of intracellular metabolites following [U-^{13}C] glucose tracing. Primary astrocytes were incubated with uniformly labeled [U-^{13}C] glucose, and intracellular metabolites were quantified by LC-MS. Labeled fractions reflect incorporation of glucose-derived carbon into downstream metabolic pathways. Bar graphs show the abundance of unlabeled (M+0) and labeled isotopologues (M+1 to M+6, indicated as C01-C06) for glycolysis-related metabolites: (**A**) lactic acid, (**B**) fructose-6-phosphate, (**C**) glucose-6-phosphate, (**D**) dihydroxyacetone phosphate, and (**E**) phosphoenolpyruvate [n = 3 biologically independent cell culture preparations; two-way ANOVA followed by Tukey post hoc test]. Data are presented as means ± SEM.

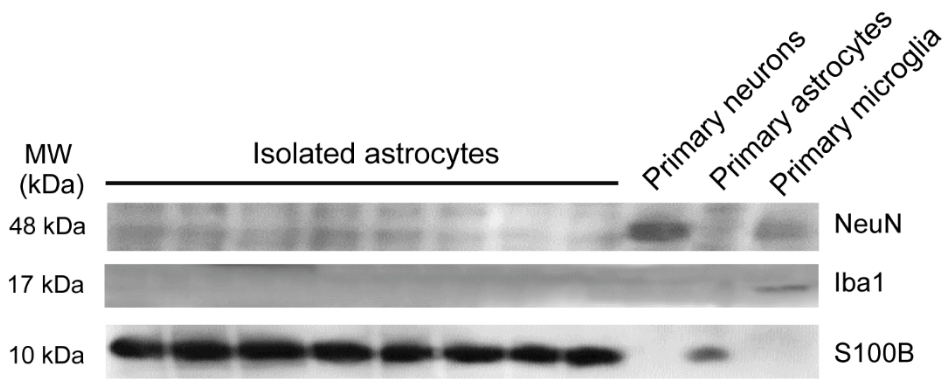

Fig. S4. Validation of the procedure for isolation of cortical astrocytes. (**A**) Representative Western blot images of NeuN, Iba1, and S100B proteins in ACSA2+ astrocytes immunomagnetically isolated from the PFC of *Npas3* cKO and control mice, primary mouse cortical neurons, primary mouse cortical astrocytes, and primary mouse cortical microglia cells.

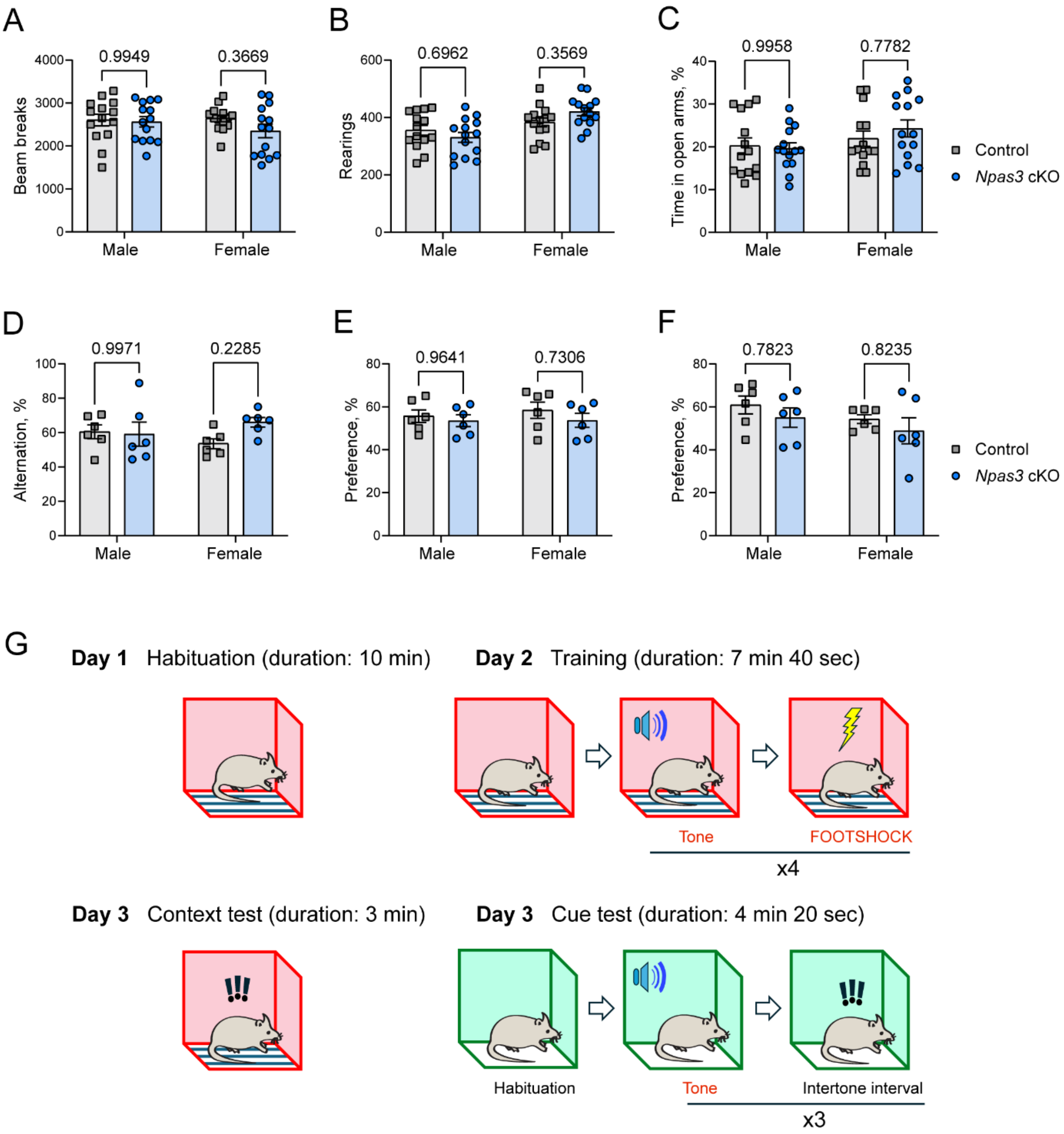

Fig. S5. No group differences in spontaneous locomotion, exploratory behavior, anxiety-related behaviors, and working memory. (**A**) Basal movement in the open field test, (**B**) rearing in the open field test, and (**C**) the preference for open arms in the elevated plus maze. Control, 7 male and 7 female mice; *Npas3* cKO, 7 male and 7 female mice. (**D**) Alternation in the Y-maze test, (**E**) the preference for novel object in the novel object recognition test (NORT), and (**F**) the preference for novel place in the novel place recognition test (NPRT). Control, 6 male and 6 female mice; *Npas3* cKO, 6 male and 6 female mice. Two-way ANOVA followed by Tukey post hoc test. Data are presented as means ± SEM. (**G**) Schematics of trace fear conditioning.

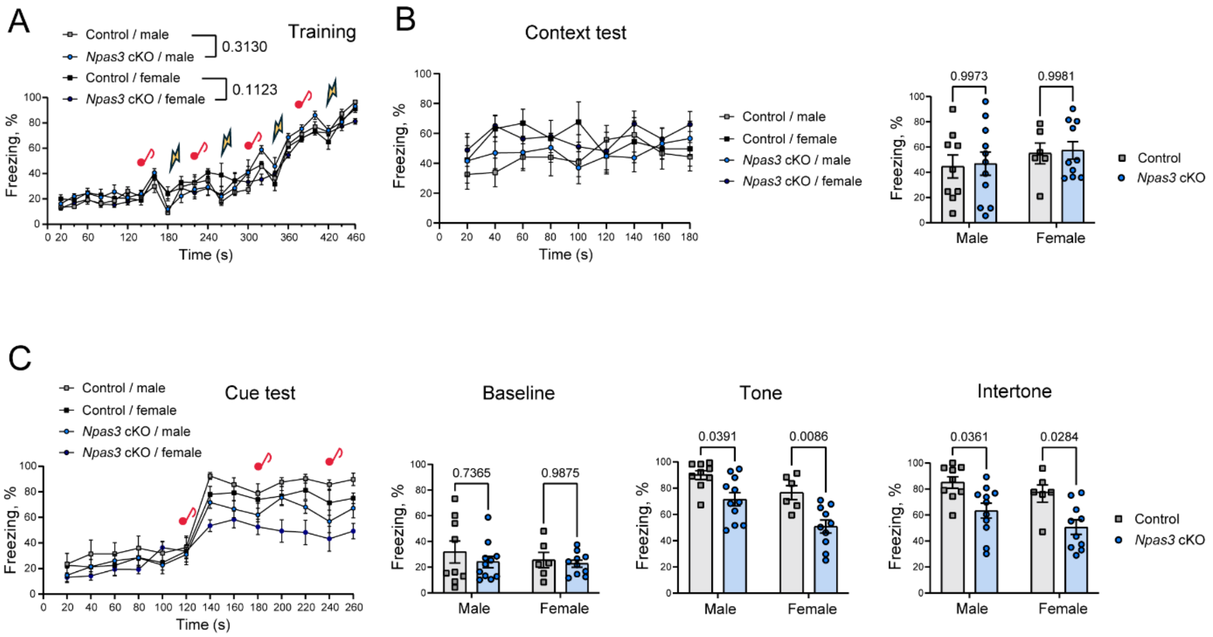

Fig. S6. No sex-dependent differences between *Npas3* cKO and control mice in TFC. (**A**) Training session freezing; repeated measures two-way ANOVA. (**B**) Context-dependent freezing over time and the average levels of context-dependent freezing. (**C**) Cue-dependent freezing measured over time, before the first tone presentation, during tone presentation, and during the inter-tone intervals. Control / male, 9 mice; Control / female, 6 mice; *Npas3* cKO / male, 11 mice; *Npas3* cKO / female, 10 mice; two-way ANOVA followed by Tukey post hoc test. Data are presented as means ± SEM.

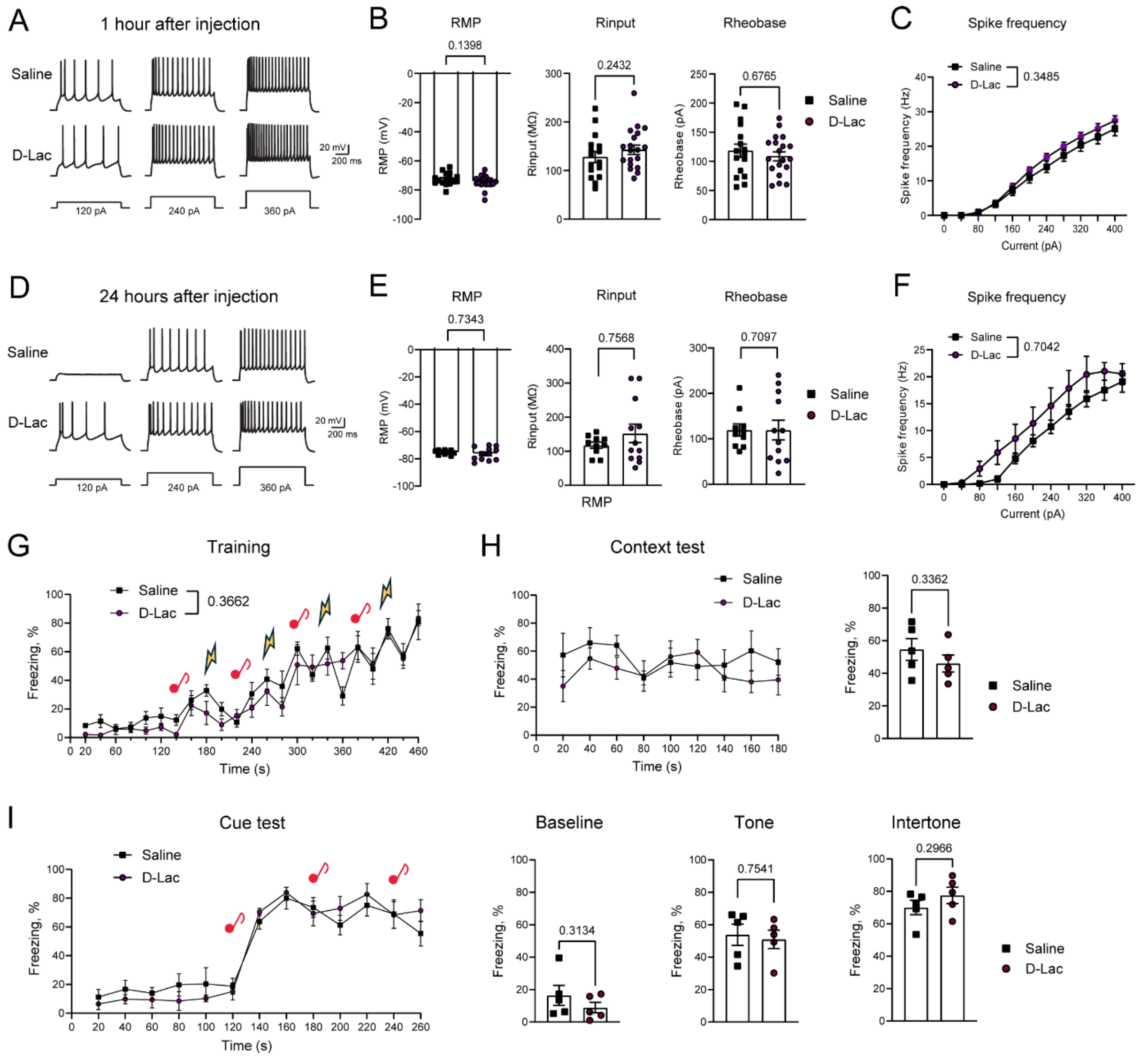

Fig. S7. D-lactate administration does not alter intrinsic excitability and TFC behavior in WT mice. (**A**) Representative voltage traces evoked by 120, 240, and 360 pA current steps measured in mPFC L5 neurons from mice injected with either saline or D-lactate (D-Lac; 1 mg/kg; i.p.) 1 h prior. (**B**) Summary graphs for resting membrane potential, input resistance, and rheobase of the neurons from mice injected with either saline or D-Lac 1 h prior [Saline, 10 cells from 3 male mice; D-lactate, 12 cells from 3 male mice; two-tailed Mann-Whitney U test]. (**C**) Current-spike frequency summary graph for the neurons from mice injected with either saline or D-Lac 1 h prior [Saline, 10 cells from 3 male mice; D-lactate, 12 cells from 3 male mice; repeated measures two-way ANOVA]. (**D**) Representative voltage traces evoked by 120, 240, and 360 pA current steps measured in mPFC L5 neurons from mice injected with either saline or D-Lac (1 mg/kg; i.p.) 24 h prior. (**E**) Summary graphs for resting membrane potential, input resistance, and rheobase of the neurons from mice injected with either saline or D-Lac 24 h prior [Saline, 10 cells from 3 male mice; D-lactate, 12 cells from 3 male mice; two-tailed Mann-Whitney U test]. (**F**) Current-spike frequency summary graph for the neurons from mice injected with either saline or D-Lac 24 h prior [Saline, 10 cells from 3 male mice; D-lactate, 12 cells from 3 male mice; repeated measures two-way ANOVA]. (**G**) Training session freezing 1 h following saline or D-Lac administration; repeated measures two-way ANOVA. (**H**) Context-dependent freezing over time and the average levels of context-dependent freezing. (**I**) Cue-dependent freezing was measured over time, before the first tone presentation, during tone presentation, and during the inter-tone intervals. Saline, 5 female mice; D-lactate, 5 female mice. Two-way ANOVA followed by Tukey post hoc test. Data are presented as means ± SEM.

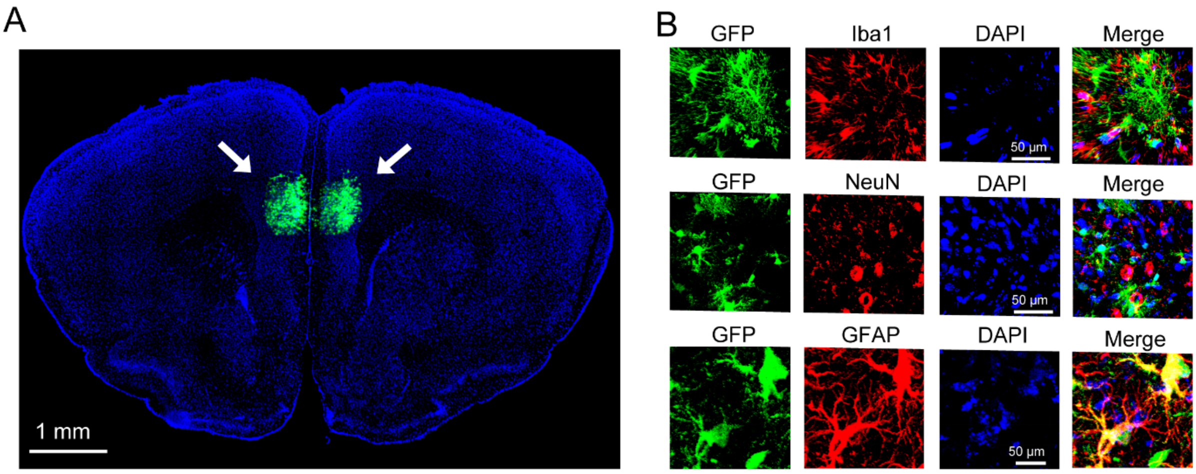

Fig. S8. Viral injection site in the mPFC and astrocyte-specific AAV transduction. (**A**) Localization of AAV in the mPFC. Representative image of a coronal section of the mouse brain with bilateral injection into the prelimbic area of the mPFC. (**B**) Verification of astrocyte-specific AAV transduction. Upper row: representative images of GFP^{+} astrocytes co-stained with anti-Iba1 (red), and DAPI (blue). Middle row: representative images of GFP^{+} astrocytes co-stained with anti-NeuN (red), and DAPI (blue). Bottom row: representative images of GFP^{+} astrocytes co-stained with anti-GFAP (red), and DAPI (blue).

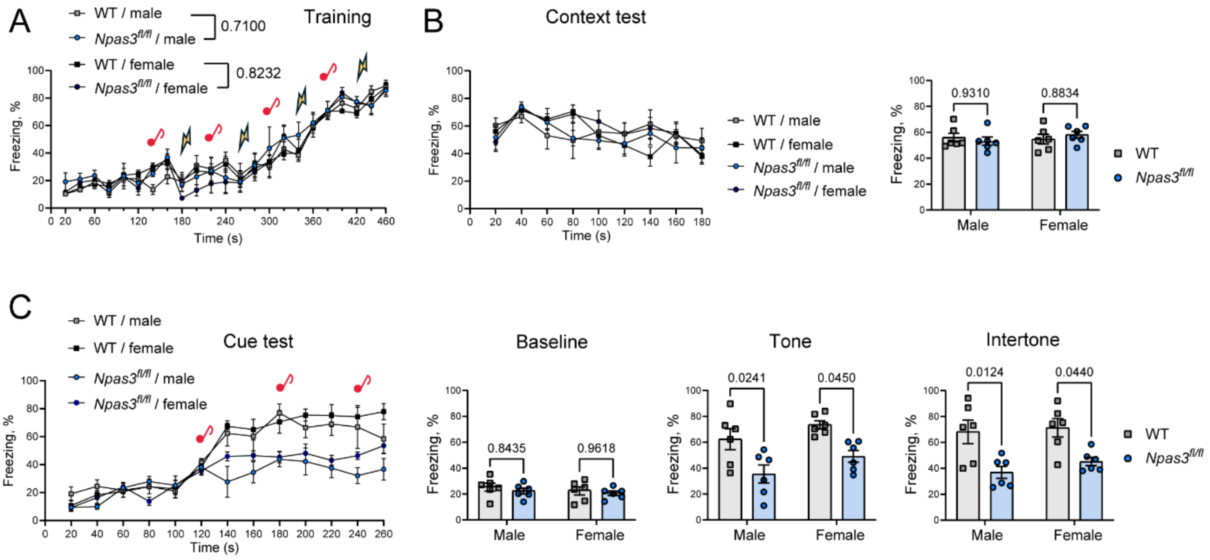

Fig. S9. No sex-dependent differences in *Npas3$^{fl/fl}$* and WT mice in trace fear conditioning. (**A**) Training session freezing; repeated measures two-way ANOVA. (**B**) Context-dependent freezing over time and the average levels of context-dependent freezing. (**C**) Cue-dependent freezing was measured over time, before the first tone presentation, during tone presentation, and during the inter-tone intervals. WT / male, 6 mice; WT / female, 6 mice; *Npas3$^{fl/fl}$* / male, 6 mice; *Npas3$^{fl/fl}$* / female, 6 mice; two-way ANOVA followed by Tukey post hoc test. Data are presented as means ± SEM.

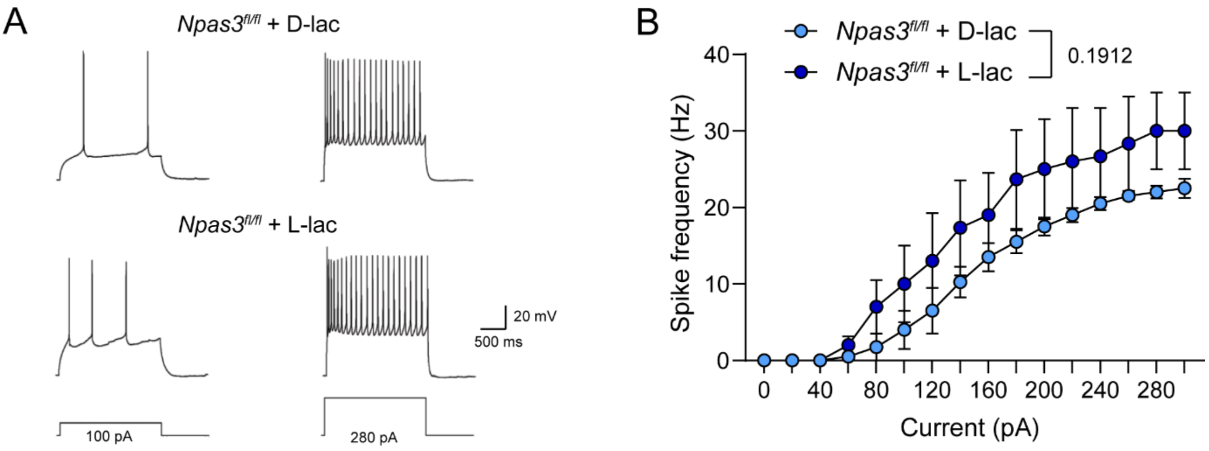

Fig. S10. L-lactate administration moderately increases excitability of mPFC neurons in *Npas3$^{fl/fl}$* mice injected with AAV-Cre. (**A**) Representative voltage traces evoked by 100 and 280 pA current steps from *Npas3$^{fl/fl}$* mice injected with either L- or D-lactate (1 mg/kg; i.p.) 24 h prior. (**B**) Current-spike frequency summary graph for L5 pyramidal neurons in mPFC [D-lactate, 4 cells from 4 male mice; L-lactate, 3 cells from 1 male mouse; repeated measures two-way ANOVA]. Data are presented as means ± SEM.

Captions for Data S1-S2

Data S1. Differential gene expression analysis results from DESeq2 modeling of forebrain tissue (E16-E17) from *Npas3*$^{+/-}$ (HET) vs. *Npas3*$^{+/+}$ (WT) female mice.

Data S2. Gene set enrichment analysis results comparing forebrain tissue (E16-E17) of *Npas3*$^{+/-}$ (HET) vs. *Npas3*$^{+/+}$ (WT) female mice for gene sets from the Mouse Molecular Signatures Database, including hallmark pathways, canonical pathways, regulatory targets, and gene ontology bioprocesses.
